# Direct Analysis in Real Time Mass Spectrometry for the Nondestructive Investigation of Conservation Treatments of Cultural Heritage

**DOI:** 10.1155/2016/6853591

**Published:** 2016-11-10

**Authors:** Marcello Manfredi, Elisa Robotti, Greg Bearman, Fenella France, Elettra Barberis, Pnina Shor, Emilio Marengo

**Affiliations:** ^1^Department of Sciences and Technological Innovation, University of Piemonte Orientale, Alessandria, Italy; ^2^ANE Image, Pasadena, CA, USA; ^3^Preservation Research and Testing Division, Library of Congress, Washington, DC, USA; ^4^Dead Sea Scrolls Digitization Projects, Israel Antiquities Authority, Jerusalem, Israel

## Abstract

Today the long-term conservation of cultural heritage is a big challenge: often the artworks were subjected to unknown interventions, which eventually were found to be harmful. The noninvasive investigation of the conservation treatments to which they were subjected to is a crucial step in order to undertake the best conservation strategies. We describe here the preliminary results on a quick and direct method for the nondestructive identification of the various interventions of parchment by means of direct analysis in real time (DART) ionization and high-resolution time-of-flight mass spectrometry and chemometrics. The method has been developed for the noninvasive analysis of the Dead Sea Scrolls, one of the most important archaeological discoveries of the 20th century. In this study castor oil and glycerol parchment treatments, prepared on new parchment specimens, were investigated in order to evaluate two different types of operations. The method was able to identify both treatments. In order to investigate the effect of the ion source temperature on the mass spectra, the DART-MS analysis was also carried out at several temperatures. Due to the high sensitivity, simplicity, and no sample preparation requirement, the proposed analytical methodology could help conservators in the challenging analysis of unknown treatments in cultural heritage.

## 1. Introduction

The conservation of cultural heritage is a challenge that could be overcome only with an interdisciplinary approach, creating new partnerships and collaborations, using new conservation technologies and promoting their diffusion.

A wide variety of analytical techniques have been applied in order to contribute to cultural heritage characterization [1] conservation and restoration, particularly on parchment [2–4]. However, practical and noninvasive methods to help curators and conservation experts are still needed. Noninvasive techniques and those requiring no sampling are always preferred.

The Dead Sea Scrolls (DSS) are a collection of about 1000 manuscripts; few are relatively well preserved, and the majority comprise thousands of fragments. These fragile manuscripts, which include the oldest existing copies of the Hebrew Bible, were preserved for two thousand years by the hot, dry desert climate and the darkness of the Judean Desert caves where they were hidden.

In the first years after they were discovered there was no awareness of their conservation needs: irreversible damage was caused by using adhesive tape for joining fragments; castor oil was lavishly spread on the fragments to enhance their reading; glycerol, BMLD (British Museum Leather Dressing, which is anhydrous lanolin, beeswax, and cedar wood oil mixed into hexane), and other unknown chemicals were used in order to preserve the scrolls. Later, the adhesive tape and some of the stains were removed with trichloroethylene and the fragments were reinforced with rice paper; polyvinyl acetate, which is of an organic glass of polymethylmethacrylate (PMMA), was used as adhesive. Other scrolls were moistened and loosely flattened between plates of windows glass without framing the edges [5].

**Figure 1:**
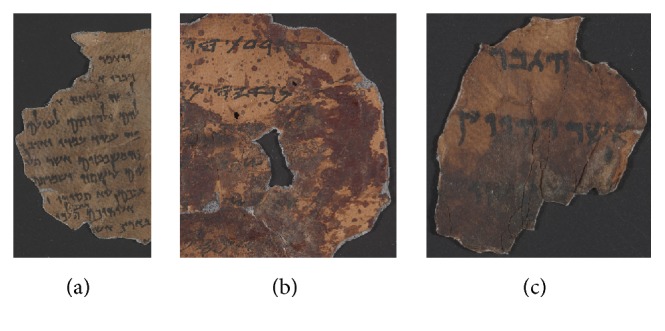
Color images of some Dead Sea Scrolls fragments: a well-preserved fragment of 4Q Deuteronomy (a), two fragments with darkening problems due to delamination—11Q Paleo Leviticus (b), and gelatinization-4Q Pesher Psalms B (c).

Over the years the adhesives penetrated the fragments and the ageing of the oils caused them further darkening so that today some of the texts are no longer legible and the edges in some cases have further gelatinized (Figure 1).

The task of preserving and conserving the DSS is crucial for their survival for future generations; thus a few years ago a project with the aim of developing a monitoring system began to regulate the conservation state of these precious manuscripts [6, 7]. But, the noninvasive investigation of the treatment to which they were subjected is a crucial step in order to undertake the best conservation and preservation strategies.

A nondestructive analytical technique that will provide fast results and identification of relevant organic compounds with no sample preparation requirements and with high sensitivity and simplicity would be of great help in the conservation and characterization of cultural heritage.

Direct analysis in real time mass spectrometry (DART-MS) is a recent MS method that identifies organic compounds in a variety of matrices quickly and directly with no need of extractions, derivatizations, and chromatographic separation. This method has already been applied in the field of cultural heritage for the characterization of paper, organic dyes, and ink [8–10]. DART is an atmospheric pressure ion source coupled with a mass spectrometer (MS) that instantaneously ionizes gases, liquids, and solids in open air under ambient conditions. The sample can be heated from room temperature to 300°C: heating is often necessary to desorb organic compounds from the sample but is obviously dangerous for the sample.

Chemometric techniques like principal component analysis, classification methods, cluster analysis, and so forth have already been applied to extract systematic information from complex dataset in the field of cultural heritage [11–13]. Statistical models were already used to classify the DART mass spectra of commercial cinnamon samples according to brand, with high specificity and low classification error [14], to rapidly determine the individual components within a psychoactive brew (Ayahuasca) made from a mixture of botanicals [15], and to identify the chemical markers of the* Rauwolfia* species [16].

The aim of this work is to develop a non-invasive method for the identification of interventions/treatments of cultural heritage using DART-MS and multivariate statistical analysis. DART-MS analysis with the ion source at room temperature was performed on castor oil and glycerol parchment treatments in order to characterize the two different classes of treatment processes.

A first principal component analysis was carried out on the DART-MS mass to charge ratio (*m*/*z*) from the treated and natural parchment (untreated) and on glycerol and castor oil standards. Then, a forward stepwise-linear discriminant analysis (FS-LDA) was performed on the principal components (PCs) calculated on the treated and untreated samples, allowing the discrimination of the classes of samples and sorting the *m*/*z* peaks according to their relevance. Linear discriminant analysis was applied to the relevant PCs for classification and identification purposes. In order to investigate the effect of the ion source temperature on the mass spectra, the DART-MS analysis was also carried out using the ion at 45°C and 90°C. Then, principal component analysis was carried out on the DART-MS *m*/*z* peaks from the treated and natural parchment (untreated) at the different temperatures.

## 2. Theory

### 2.1. PCA and LDA

PCA [17] is a multivariate pattern recognition method representing the objects, described by the original variables, into a new reference system given by new variables called PCs, linear combinations of the original variables. PCs are orthogonal one to each other and are calculated hierarchically in decreasing order of explained variance so that experimental noise and random variations are collected in the last PCs. PCA offers therefore a powerful tool for dimensionality reduction. PCA provides two tools for data analysis: the* scores* (the coordinates of the objects on the PCs), allowing the identification of groups of samples showing similar or opposite behavior, and the* loadings* (the weights of the original variables on each PC), giving insight on the reasons of the differences pointed out in the objects. PCA has already been applied to separate systematic information from experimental noise and to describe the conservation treatments in a compact and efficient way by using the more significant PCs [18].

LDA is a Bayesian classification method providing the classification of the objects considering the multivariate structure of the data [19]. In Bayesian methods each class is usually described by a Gaussian multivariate probability distribution and each object is assigned to a particular class if the so-called discriminant score is minimum; it is calculated by

(1) $$\begin{array}{c} D\left(\;g\;|\;x\;\right)={\left(\;x_{i}-c_{g}\;\right)}^{T}S_{p}^{-1}\left(\;x_{i}-c_{g}\;\right)+\ln\left|\;S_{p}\;\right|-2\ln P_{g}, \end{array}$$

where (*x*_*i*_ − *c*_*g*_)^*T*^ *S*_*g*_^−1^(*x*_*i*_ − *c*_*g*_) is the Mahalanobis distance between the *i*th object and the centroid of class *g*, *S*_*p*_ is the class covariance matrix (that is approximated with the pooled covariance matrix between classes), and *P*_*g*_ is the prior probability of class *g*, set here equal for all classes. The classification performance of the LDA models can be evaluated by the calculation of the nonerror rate (NER%) that represents the percentage of overall correct assignments. The prediction performance of the models can be evaluated by cross-validation techniques. The variables to be included in the LDA model can be chosen by a stepwise algorithm that selects the most discriminating variables. The procedure adopted here exploits a forward search (FS) algorithm: at each iteration a variable is added to the model according to the highest NER% in cross-validation. In this study, due to the large number of variables present, FS-LDA was applied to principal components rather than to the original variables: PCA was therefore used as dimensionality reduction tool providing a set of descriptors (PCs) summing up systematic information in a restricted set of PCs.

The validation of the LDA model was performed as follows:

1. The samples were divided into training set and test set, including in the test set the 20% of the samples of each class.
2. PCA was applied to the samples belonging to the training set and samples from the test set were reprojected in the space given by the PCs calculated.
3. FS-LDA was applied to the training set selecting at each iteration the PC to be included in the model according to the highest increase in the NER% calculated in cross-validation. Cross-validation was applied by bootstrapping with 1000 iterations excluding from the training set the 20% of the samples.
4. The test set was used to evaluate the prediction performance of the LDA model.

Steps from (1) to (4) were repeated 100 times selecting each time a different test set by random sampling. Here, we present the average results obtained on the 100 random samplings performed; the final model presented represents the overall best solution.

## 3. Experimental

### 3.1. DART-MS

The analyses were conducted with a JEOL AccuTOF mass spectrometer (JEOL USA, Peabody, MA) equipped with a DART ion source (Ionsense, Saugus, MA) in positive ion mode, with helium being the ionization gas under the following conditions: flow rate of 2.5 L/min, gas temperature at 23°C, 45°C, and 90°C, and grid voltage of +350 V. Orifice 1 was held at 120°C and 30 V, orifice 2 was at 5 V, and the ring lens voltage was set to 5 V. The peaks voltage was held at 1500 V and the instrument was calibrated with a solution of PEG 600 in methanol. The mass spectrum recording interval was 1.00 s and the *m*/*z* values were from 50 Da to 800 Da. The mass resolution was approximately 6000. The ceramic outlet of the ion source was positioned 15 mm away from the pinhole orifice that leaks into the mass analyzer. All equipment in contact with the parchment samples was cleaned using rinses of purified water and acetone.

### 3.2. Parchment Samples

Parchment is a semitanned skin used as a writing surface. Of all the components that constitute the living skin, only insoluble proteins and water remain when this is transformed into a parchment.

Vegetable adhesives like flour paste, synthetic polymers (polyvinyl acetate (PVA) solutions), and acrylic resin solutions were widely used in the past in order to support the parchments. Sometimes parchments were lubricated with organic materials like glycerol, petroleum jelly, lanolin, oils, or other very different substances in a misguided attempt to “condition” the parchments and to enhance their readability. The use of lubricants caused alteration of the surface texture, increase of transparency, attraction of surface dirt, and darkening the skin. The use of unknown organic solvents caused damage to the collagen structure.

Glycerol, which is a simple polyol compound, and castor oil, a vegetable oil obtained from the castor bean, were often used in the past for the lubrication of horny parchments.

For this research we used a goat parchment made by an Orthodox Rabbi from Jerusalem in 2009. The parchment was prepared in compliance with rabbinical rules. Castor oil and glycerol parchment treatments were investigated in order to evaluate two different intervention processes that are suspected to have been applied to the Dead Sea Scrolls in the past and to be the cause of some of their evident deteriorations. Two separate parchment samples were spread with commercial castor oil (CVS, Woonsocket, Rhode Island, USA) and glycerol (Sigma-Aldrich, St. Louis, Missouri, United States). The samples were left for 48 hours at room temperature and were then analyzed by DART-MS.

### 3.3. Software

The statistical analysis, the spectral alignment, and the graphical representations were performed by The Unscrambler X (CAMO PROCESS, AS, Oslo, Norway), home-made routines built in MATLAB R2014a environment (The Mathworks, Inc., Natick, MA, USA), and the Classification Toolbox for MATLAB from the Milano Chemometrics Group [20].

### 3.4. Methodology

Parchment samples were cut into 5 × 20 mm strips using clean scissors; then, they were positioned at room temperature in helium atmosphere at a distance of 1 mm from the ceramic outlet of the DART ion source by using fine-point steel tweezers. No sample preparation was necessary.

Castor oil and glycerol standards were analyzed by dipping a melting point tube into the sample and placing the tube in front of the DART ion source for a few seconds.

## 4. Results and Discussion

The aim of the research was the development of a nondestructive methodology for the analysis of unknown interventions of parchment by using DART-MS.

The temperature of helium significantly impacts the information obtained by DART-MS: the substances detected by the instrument strongly depend on the temperature of the carrier gas in the ion source.

Parchment is an organic material mainly made of collagen and water: temperature is one of its worst enemies. We performed the analysis using the flux of helium at room temperature in order to avoid causing any damage to the parchment surface.

**Figure 2:**
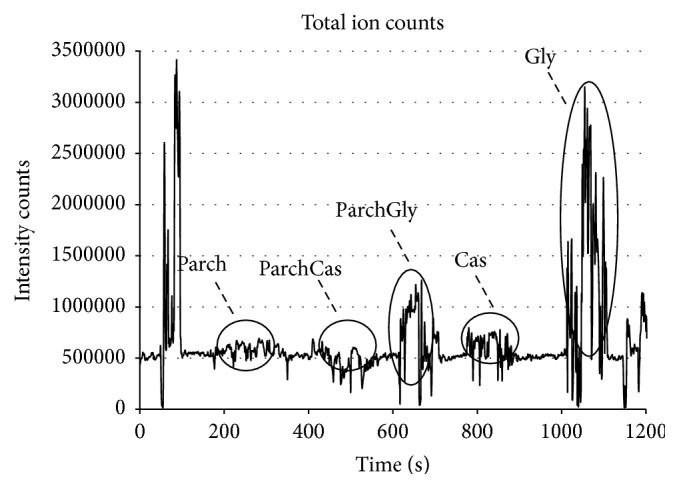
Total ion chromatogram (intensities of all mass spectral peaks belonging to the same scan) of analyzed samples: from time 230 to 300 seconds an untreated parchment sample was positioned in the helium stream; from time 500 to 550 seconds a piece of parchment treated with castor oil was analyzed; from time 630 to 700 seconds a piece of parchment treated with glycerol was analyzed; from time 780 to 880 seconds a standard of castor oil was inserted in the gap and analyzed; form time 1020 to 1100 seconds a standard of glycerol was positioned in the helium flux and analyzed.

We analyzed an untreated parchment sample, a parchment sample treated with castor oil, a parchment sample treated with glycerol, and castor oil and glycerol standards as shown by the total ion chromatogram (TIC) (Figure 2).

**Figure 3:**
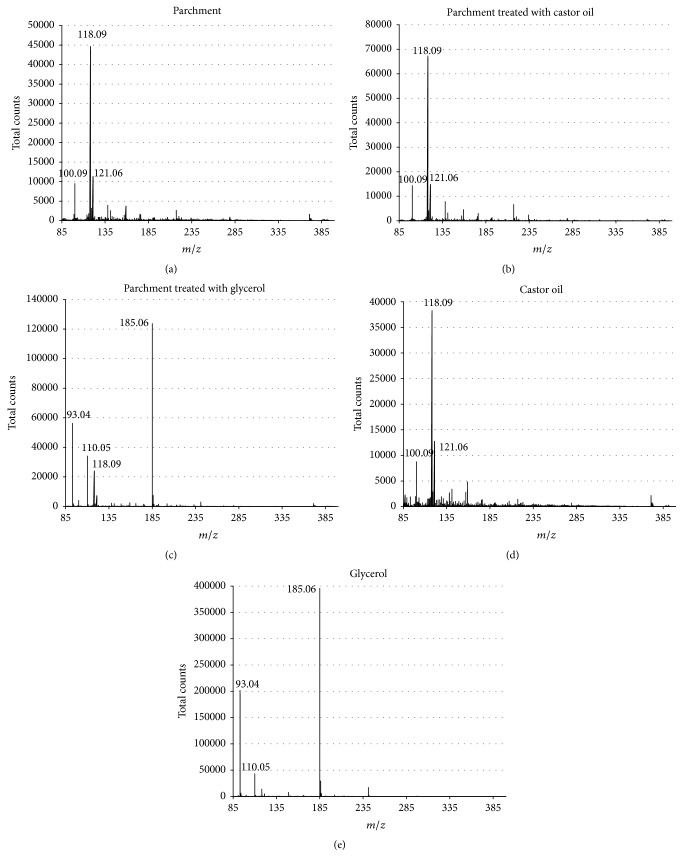
(a) Mass spectrum of untreated parchment, (b) mass spectrum of parchment treated with castor oil, (c) mass spectrum of parchment treated with glycerol with the signal at *m*/*z* 93.049 (protonated: M+H+) and 185.063 (dimeric ions: M+M+H+), (d) mass spectrum of castor oil standard, and (e) mass spectrum of glycerol standard.

Although the carrier gas in the ion source temperature was set at 23°C, low-mass products (≈80 to 400 *μ*), generated by the impact of the carrier gas on the surface of the samples, from untreated and treated parchment and from castor oil and glycerol, are clearly identifiable. Figures 3(a), 3(b), 3(c), 3(d), and 3(e) show, respectively, the mass spectrum of untreated parchment, parchment treated with castor oil, and parchment treated with glycerol, castor oil standard, and glycerol standard.

The *m*/*z* of fragments of untreated parchment and parchment treated with castor oil and castor oil are not clearly assignable because of the complexity of the matrix and of the low working temperature. Moreover the three mass spectra are very similar to each other because both parchment and castor oil contains lipid groups.

The mass spectra of parchment treated with glycerol and of standard glycerol (Figures 3(c) and 3(e)) show that *m*/*z* 93.049 (M+H+) and 185.063 (M+M+H+) are major signals and correspond, respectively, to protonated glycerol and protonated glycerol clusters: the dimeric signal is probably due to the higher analytes concentration. The *m*/*z* 110 corresponds to the glycerol water adduct (M+H20). The glycerol treatment on parchment is recognizable by looking at the mass spectrum: the characteristic *m*/*z* 93, 110, and 185 of glycerol are clearly present. While looking at the mass spectra of parchment treated with castor oil, it is not possible to identify the treatment because parchment and castor oil have a similar mass spectrum profile. This is due to the low temperature of the ion source (*T* = 23°C) employed for the analysis: the ionization and the formation of product ions are more difficult for castor oil and parchment. Several studies showed that the heater temperature affects the fragmentation and the signal of the analytes. In order to identify unknown parchment treatment without interpreting mass spectrum, especially when the interpretation is not easy to perform because of the complexity of the spectra, we performed a multivariate statistical analysis on the data collected by DART-MS.

### 4.1. Statistical Analysis and Variable Selection

#### 4.1.1. Principal Component Analysis on Overall Dataset

**Figure 4:**
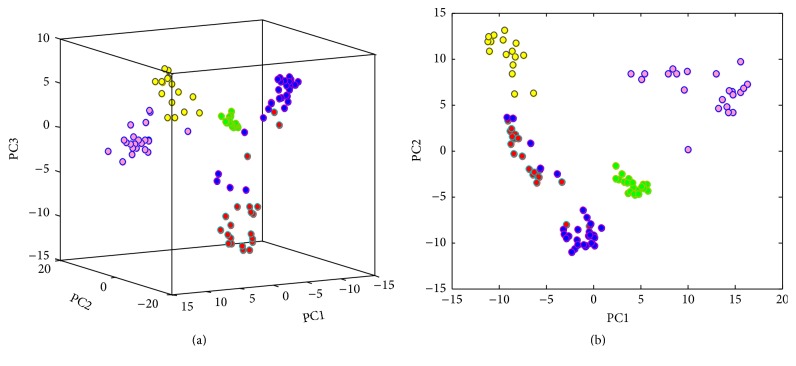
Score plot of PC1, PC2, and PC3 (a). The samples are separated along the three PCs in five clusters: untreated parchment sample (red), the parchment sample treated with castor oil (blue), the parchment sample treated with glycerol (green), the castor oil standard (yellow), and the glycerol standard (pink). (b) represents the score plot of PC1 and PC2.

The mass spectra of the different samples matched each other and the results were arranged in a 106 × 6346 matrix: 106 being the number of samples (19 parchment samples, 28 parchment samples treated with castor oil, 21 parchment samples treated with glycerol, 16 samples of castor oil, and 22 samples of glycerol) and 6346 being the *m*/*z* intensity count for each sample. Samples were first-rowwise range scaled between 0 and 1 to eliminate possible effects due to differences in the amount of sample used to record each spectrum. Then the dataset was columnwise range scaled between 0 and 1 before performing PCA. The amount of explained variance is distributed along several PCs showing a relatively low correlated data structure (PC1 = 6.9%, PC2 = 5.9%, PC3 = 3.5%, PC4 = 2.4%, and PC5 = 1.7%). The low correlation is explained by significant sample heterogeneity. In the score plot of PC1 versus PC2 versus PC3 (Figure 4(a)) the samples are well separated along the three PCs in five clusters that represent the untreated parchment sample (red), the parchment sample treated with castor oil (blue), the parchment sample treated with glycerol (green), the castor oil standard (yellow), and the glycerol standard (pink).

The first component explains the differences between untreated parchment (red) and the treated samples (blue, green, yellow, and pink); the second component explains the differences between the parchment samples (red, blue, and green) and the standard samples (yellow, pink) as is shown in Figure 4(b).

#### 4.1.2. Principal Component Analysis on Parchment Samples

Since the main target was the identification of unknown parchment treatments, the analysis was restricted to parchment samples in order to find the most significant PCs useful to discriminate the conservation treatments.

**Figure 5:**
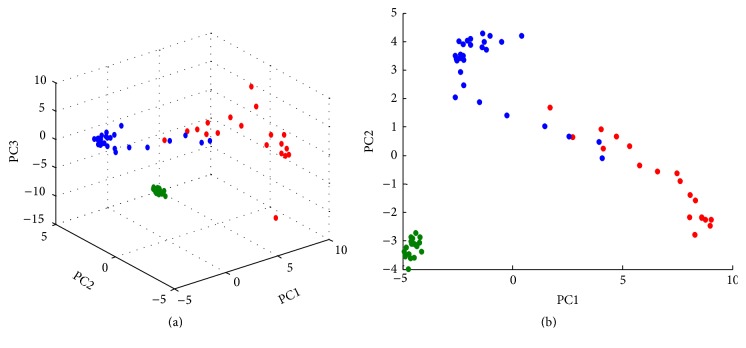
Score plot of PC1, PC2, and PC3 (a). The samples are separated along the three PCs in three clusters: untreated parchment sample (red), the parchment sample treated with castor oil (blue) and the parchment sample treated with glycerol (green). (b) represents the score plot of PC1 and PC2.

PCA was then applied to the restricted set of data: the spectra were arranged in a 68 × 3987 matrix: 68 being the number of samples (19 parchment samples, 28 parchment samples treated with castor oil, and 21 parchment samples treated with glycerol) and 3987 being the *m*/*z* intensity count for each sample. PCA was carried out after rowwise range scaling between 0 and 1 followed by columnwise range scaling between 0 and 1. The amount of explained variance is quite distributed along several PCs (PC1 = 15.99%, PC2 = 6.07, PC3 = 3.50%, PC4 = 3.38%, and PC5 = 3.27%). In the score plot of PC1 versus PC2 versus PC3 (Figure 5(a)) the samples are well separated along the three PCs in three clusters that represent the untreated parchment sample (red), the parchment sample treated with castor oil (blue), and the parchment sample treated with glycerol (green). The first component explains the differences between untreated parchment (red) and the conservation treatment (blue and green) and the second component explains the differences between the two different conservation treatments: castor oil (blue) and glycerol (green) as is shown in Figure 5(b).

PCA allows reducing the variable dimensionality: the latest PCs, mainly accounting for noise and experimental error, are not considered further in this study. The score plot shows that the parchment samples treated with glycerol are characterized by a small dispersion; untreated parchment and parchment treated with castor oil are characterized by a quite large dispersion: this can be explained by the complexity of the analyzed matrix of the samples that is difficult to ionize using the helium stream at room temperature.

Regarding the separation between classes, the score plot shows that the samples are quite well separated: this is particularly true for the samples treated with glycerol.

Instead, some untreated parchment samples overlapped with the samples treated with castor oil and vice versa: this can be explained by the low temperature of analysis that limits the ionization products of the two samples.

#### 4.1.3. Linear Discriminant Analysis

The identification of possible markers, which are able to discriminate the conservation treatment, was then achieved by linear discriminant analysis applied to the first 20 PCs calculated: the use of the PCs instead of the original variables allows the dimensionality reduction and the elimination of the experimental noise. The data were divided into training and test sets: the test set included the 20% of the samples of each class (14 samples: 4 samples from untreated and glycerol treated parchment and 6 samples from castor oil treated parchment). PCA was applied to the samples belonging to the training set and samples from the test set were reprojected in the space given by the first 20 PCs calculated. FS-LDA was then applied selecting the significant PCs by cross-validation (bootstrapping with 1000 iterations). The overall procedure was repeated 100 times and the samples in each different test set were randomly selected from the overall dataset, as described in the theory section.

Table 1 reports the results obtained for the 100 test sets used: the models contained from a minimum of 2 PCs to a maximum of 8 PCs. All the models showed very good NER% ranges both on the training set (from 96.30% to 100%), in cross-validation (from 93.48 to 99.52%), or on the test set (from 85.71% to 100%).

**Figure 6:**
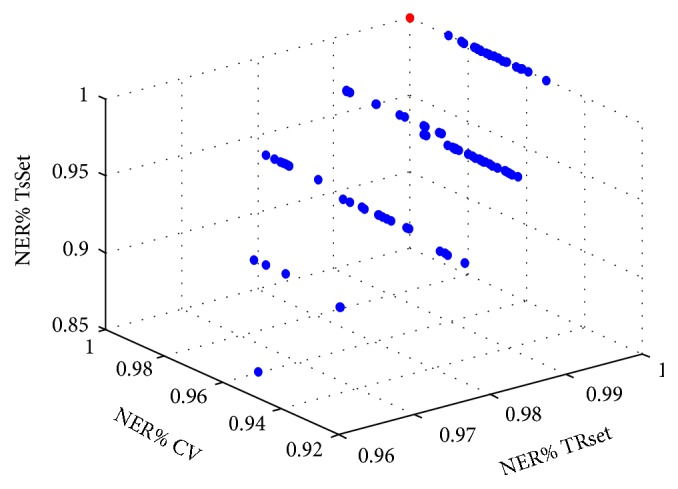
Results for the 100 models calculated. *x*-axis: NER% calculated on the training set; *y*-axis: NER% calculated in cross-validation; *z*-axis: NER% calculated on the test set. The best theoretical model corresponding to a NER equal to 100% on the three axes is indicated in red.

Figure 6 represents the results obtained for the 100 models calculated representing on the *x*-axis the NER% on the training set, on the *y*-axis the NER% in cross-validation, and on the *z*-axis the NER% on the test set. The figure also reports the best theoretical model corresponding to a NER equal to 100% for all the three sets of samples (indicated in red in the figure); the overall best calculated model was selected as the closest to the best theoretical one. Figure 6 shows that the 100 models calculated show very good results, providing good performances not only on the training set but also in prediction, proving the robustness of the results obtained.

**Figure 7:**
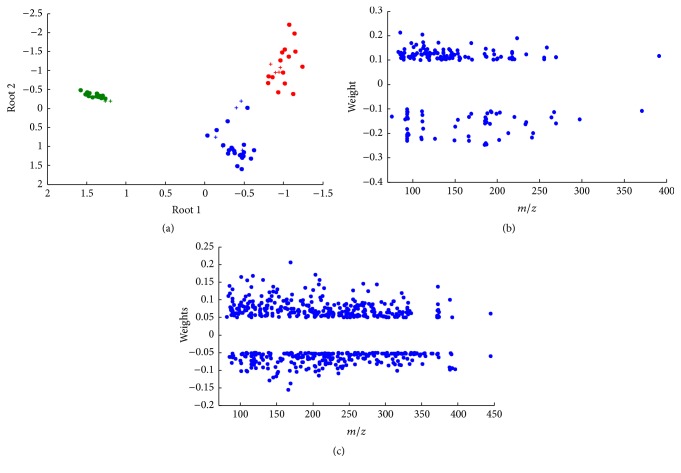
Results of canonical analysis: canonical scores of Root 1 and Root 2 (a). The samples are separated along the two roots in the three classes: untreated parchment sample (red), the parchment sample treated with castor oil (blue), and the parchment sample treated with glycerol (green). Circles represent samples from the training set while crosses represent samples from the test set. (b) and (c) represent the weights of the original variables on Root 1 and Root 2, respectively: original variables are reported on the *x*-axis and the weights on the *y*-axis. Only the most significant weights are reported (identified by normal probability plots).

The overall best model provided very good results in calibration and prediction: all samples from the training and test sets were correctly assigned (NER and specificity of 100% for all classes) and no overlap was detected (selectivity of 100% for all classes). The results are slightly worse in cross-validation, with a NER% equal to 98.68%. The model includes 6 PCs: PC1, PC2, PC9, PC10, PC12, and PC15.  Table 2 reports the coefficients of each PC included in the LDA classification model on the first two canonical roots calculated by canonical analysis. This result is graphically represented in Figure 7(a), reporting the samples along the two discriminant roots calculated by canonical analysis: circles correspond to samples belonging to the training set and crosses represent samples from the test set, while color indicates the class. The first root separates glycerol treated samples (at negative values) from the other two classes (at positive values), while the second root separates untreated samples (at positive values) from castor oil treated samples (at negative values). The figure clearly shows the perfect classification of the samples from both training and test sets, since the three groups of samples are well separated.

Since the PCs are linear combinations of the original variables, it is possible to calculate the weight of each original variable (*m*/*z* signal) on the final model and to identify the variables characterized by the most significant contribution (i.e., markers). The results are reported in Figures 7(b) and 7(c) separately for the two canonical roots identified: the original variables are represented on the *x*-axis and the weight on the corresponding root is reported instead on the *y*-axis; only the variables characterized by a significant weight were represented (the significant weights being identified by normal probability plots [17]). Negative weights on root 1 correspond to signals characterized by a large intensity in glycerol treated samples and a small signal in the other two classes, while positive weights correspond to signals with an opposite behavior. Positive weights on root 2 correspond instead to signals more intense in untreated samples (and in a minor way in glycerol treated samples) and characterized by a small intensity in castor oil treated samples, while negative weights show an opposite behavior. The complete list of the calculated coefficients is reported in Supplementary Information 1 of the Supplementary Material available online at http://dx.doi.org/10.1155/2016/6853591.

#### 4.1.4. Effect of the Working Temperature

The effect of the ion source temperature on the mass spectra has been investigated by analyzing the treated parchment samples and the natural parchment samples (untreated) using several source (carrier gas) temperatures: 23°C, 45°C, and 90°C.

The mass spectra of the different samples matched each other and the results were arranged in a 232 × 5323 matrix: 232 being the number of samples (69 samples analyzed at 23°C, 78 samples analyzed at 45°C, and 85 samples analyzed at 90°C) and 5323 being the *m*/*z* intensity count for each sample. PCA was carried out after range scaling between −1 and 1. The amount of explained variance is distributed along several PCs showing a relatively low correlated data structure (PC1 = 10.1%, PC2 = 7.4%, PC3 = 6.5%, PC4 = 3.5%, and PC5 = 1.7%).

For each working temperature it is possible to identify groups corresponding to the different types of treatment: natural parchment (star), parchment treated with glycerol (circle), and parchment treated with castor oil (diamond). The position of the clusters changes with temperature, with the exception of the samples treated with glycerol that remains approximately in the same position at all temperatures.

**Figure 8:**
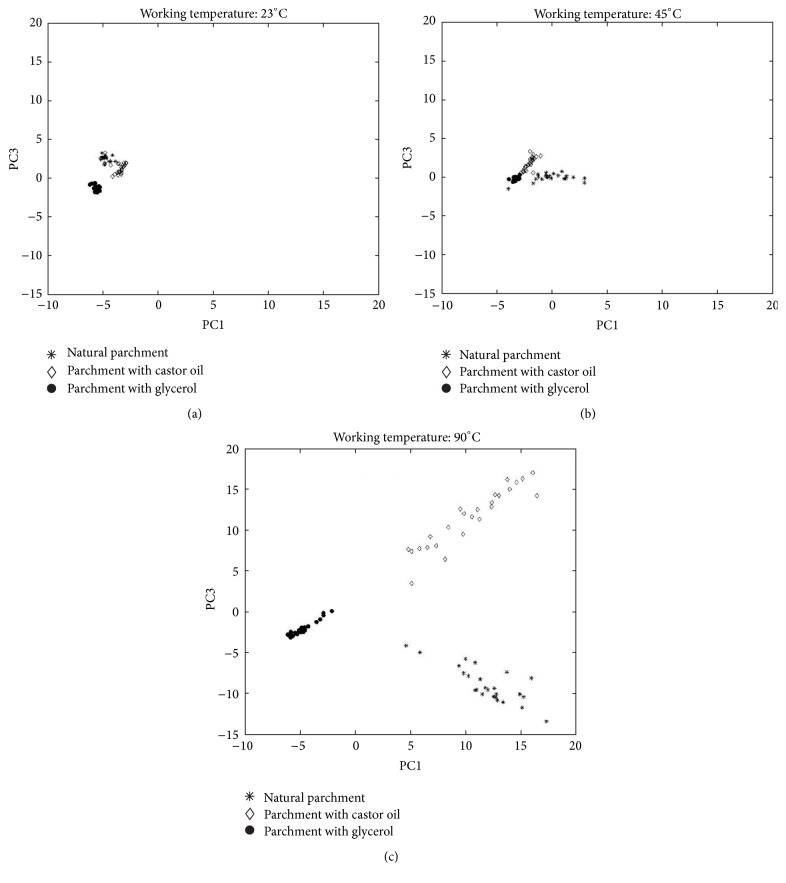
Score plots of PC1 and PC3 for each working temperature and for the 3 different groups of samples.

At lower working temperature (Figures 8(a) and 8(b)) the groups of samples are separated but are closer to each other and more homogeneous within the same group: increasing the temperature causes an increase of the distance between the 3 groups of samples in the PC reference system and a spread of the samples within each group. In particular the scores of natural parchment samples (star) and parchment samples treated with castor oil (diamond) move towards larger values.

In the score plot of PC1 versus PC3 (Figure 8(c)) the first principal component explains mainly the effect of the temperature at 90°C: at high scores there are the natural parchment samples (Figure 8(c), star) and the parchment samples treated with castor oil (Figure 8(c), diamond) analyzed at 90°C. Parchment samples treated with glycerol (circle) at 23°C, 45°C, and 90°C are characterized by a smaller dispersion: the mass spectra of these samples do not significantly change working at different temperature, probably since glycerol is volatile enough at room temperature to obtain good mass spectra also in the least extreme conditions.

PC3 separates the samples into three clusters: untreated parchment samples (star), parchment samples treated with castor oil (diamond), and parchment samples treated with glycerol (circle), all analyzed at 90°C (Figure 8(c)); all the parchment samples treated with glycerol (circle) analyzed at 23°C (Figure 8(a)), 45°C (Figure 8(b)), and 90°C (Figure 8(c)) are grouped together and have a low dispersion natural parchment samples (star) and parchment samples treated with castor oil (diamond) at 23°C (Figure 8(a)) and at 45°C (Figure 8(b)) have a similar pattern and are not well separated: increasing the temperature causes an increase of the distance between the 3 groups of samples in the PC reference system.

The third principal component mainly explains the differences between the natural parchment samples (Figure 8(c), star) and parchment samples treated with castor oil (Figure 8(c), diamond) at 90°C.

Increasing the carrier temperature the identification of different parchment treatment is easier because the gas ionizes also the less volatile molecules. Unfortunately this condition is not feasible for the analysis of the original Dead Sea scrolls, as using high temperature makes the analysis destructive.

## 5. Conclusions

In this study we developed a new noninvasive method for the identification of unknown conservation treatments of cultural heritage, in particular for parchment by using DART-MS and statistics.

Many traditional interventions can result in irreversible alteration of an artifact; because of its animal origin, parchment might respond to treatments in unpredictable ways. The conservator's approach to the treatment of parchment must be extremely cautious and, due to the simplicity and no sample preparation requirement, the proposed analytical tool could help in the challenging analysis of unknown treatments in cultural heritage.

Castor oil and glycerol parchment treatments were investigated using the DART carrier gas in the ion source at room temperature in order not to cause any damage to parchment samples: this is very important while working with any cultural heritage and even more with the Dead Sea Scrolls which are extremely fragile and precious.

The method was able to identify both treatments: FS-LDA performed on principal components revealed to be a robust tool that could be employed for the classification of unknown treatments, overall in presence of samples with similar mass spectrum profiles.

While this study looked at a small number of conservation treatments, there is additional work that could be completed. A DART-MS library of treatments may be created and extended including also the degradation products that may appear after the ageing of the object. The technique may also be applied other cultural heritage objects, such as paintings and other artifacts. Moreover, the investigation of the working temperature of the ion source could help in the identification of unknown substances because at higher temperature the ionization of molecules is easier, even if the analysis could become destructive.

Several studies published the possibility of using different geometry/configuration of the DART [21]: the 45° configuration can be used to analyze or scan surfaces. This configuration allows the analysis of bigger samples like parchment fragments and manuscripts of the Dead Sea Sea Scrolls: our study confirmed that the method can be employed in a noninvasive manner and the next step will be the analysis of the scrolls using this configuration.

In conclusion, using chemometrics, we are able to identify unknown parchment treatment without using high temperature, preserving the integrity and the state of conservation of the samples.

## Supplementary Material

Weights of each original variable on the first and second Roots calculated by canonical analysis. For Root 1, weights with absolute value > 0.1 were considered statistically significant; for Root 2 instead, statistically significant weights were characterized by an absolute value > 0.05. Statistically significant weights were identified by Normal Probability plots.

## Figures and Tables

**Table 1:** Results of LDA on the training and test sets and in cross-validation calculated on the 100 test sets.

Number of PCs included		NER% training set		NER% cross-validation		NER% test set	
Min	Max	Min	Max	Min	Max	Min	Max
2	8	96.30	100	93.48	99.52	85.71	100

**Table 2:** Coefficients of the significant PCs included in the final LDA model on the first two canonical roots calculated.

	Loadings on Root 1	Loadings on Root 2
PC1	0.7030	−0.6584
PC2	0.7108	0.6446
PC9	0.0092	0.0949
PC10	−0.0104	0.1236
PC12	−0.0102	−0.2988
PC15	−0.0107	−0.1933
