# Supplementary material for: Direct Analysis in Real Time Mass Spectrometry for the Nondestructive Investigation of Conservation Treatments of Cultural Heritage
Source: J Anal Methods Chem. 2016 Nov 10;2016:6853591. doi: 10.1155/2016/6853591 (PMC5121580; doi:10.1155/2016/6853591)
Supplement: Supplementary file 1 — Weights of each original variable on the first and second Roots calculated by canonical analysis. For Root 1, weights with absolute value > 0.1 were considered statistically significant; for Root 2 instead, statistically significant weights were characterized by an absolute value > 0.05. Statistically significant weights were identified by Normal Probability plots. [file 6853591.f1.pdf]

Title: Direct Analysis in Real Time Mass Spectrometry for the Non-destructive Investigation of Conservation Treatments of Cultural Heritage

Authors: Marcello Manfredi*[1], Elisa Robotti[1], Greg Bearman[2], Fenella France[3], Elettra Barberis[1], Pnina Shor[4], Emilio Marengo[1]

Supporting Information 1. Weights of each original variable on the first and second Roots calculated by canonical analysis. For Root 1, weights with absolute value > 0.1 were considered statistically significant; for Root 2 instead, statistically significant weights were characterized by an absolute value > 0.05. Statistically significant weights were identified by Normal Probability plots.

m/z	Weight on Root 1	Weight on Root 2	m/z	Weight on Root 1	Weight on Root 2	m/z	Weight on Root 1	Weight on Root 2
**75.037**	-0.131	0.027	**188.078**	0.084	0.066	**259.056**	-0.011	-0.053
**81.058**	0.021	-0.028	**188.086**	0.009	-0.016	**259.062**	0.012	0.017
**81.063**	0.072	0.052	**188.091**	0.018	-0.049	**259.077**	0.003	-0.016
**83.039**	0.018	0.020	**188.1**	0.024	0.017	**259.125**	-0.012	0.005
**83.044**	0.012	0.017	**188.106**	0.035	-0.042	**259.133**	-0.008	0.002
**83.073**	0.133	0.111	**188.115**	0.034	-0.001	**259.14**	0.012	-0.023
**83.078**	-0.005	-0.038	**188.123**	0.030	0.031	**259.147**	0.056	0.076
**85.018**	0.213	0.047	**188.131**	-0.002	0.017	**259.155**	0.017	0.020
**85.052**	0.124	0.139	**188.147**	0.025	0.013	**259.164**	0.017	-0.049
**85.057**	-0.008	-0.062	**188.155**	0.025	0.029	**259.176**	-0.005	-0.010
**85.072**	0.013	0.002	**188.163**	0.016	-0.016	**259.185**	0.007	-0.016
**85.078**	0.007	-0.001	**188.171**	0.002	-0.030	**259.195**	0.006	-0.004
**85.086**	0.070	0.119	**188.18**	0.008	-0.004	**259.214**	-0.012	0.003
**85.091**	0.011	-0.022	**188.195**	0.025	-0.006	**259.223**	-0.014	0.004
**85.099**	0.010	-0.006	**188.212**	0.056	0.043	**259.286**	0.006	-0.002
**85.132**	0.010	0.003	**188.243**	0.018	0.020	**260.06**	-0.013	0.001
**86.05**	0.131	0.079	**189.042**	0.001	-0.046	**260.069**	0.066	0.012
**86.055**	0.011	-0.013	**189.052**	0.006	0.006	**260.081**	0.010	0.003
**86.083**	0.109	0.033	**189.063**	0.060	0.134	**260.098**	0.011	0.030
**86.09**	0.004	-0.008	**189.072**	0.003	0.029	**260.105**	0.036	0.055
**86.107**	-0.010	-0.007	**189.082**	-0.032	-0.040	**260.111**	0.034	0.026
**86.112**	0.015	-0.062	**189.089**	0.033	-0.022	**260.12**	0.017	-0.034
**86.128**	0.007	-0.009	**189.094**	0.010	-0.010	**260.13**	0.005	-0.004
**86.134**	-0.012	-0.042	**189.1**	-0.022	-0.052	**260.14**	0.044	0.020
**86.139**	0.007	-0.008	**189.12**	-0.014	0.002	**260.149**	0.030	-0.011
**86.147**	0.024	0.019	**189.136**	0.003	-0.009	**260.156**	0.021	-0.026
**86.166**	0.020	0.012	**189.145**	0.006	-0.011	**260.162**	0.041	0.034
**87.033**	0.112	0.076	**189.177**	0.021	0.034	**260.173**	-0.020	0.025
**87.06**	0.064	0.071	**189.193**	0.015	0.002	**260.187**	0.032	0.002
**87.066**	0.042	0.034	**189.202**	0.012	0.017	**260.195**	0.022	-0.018
**87.071**	-0.005	-0.029	**189.209**	0.017	0.015	**260.205**	0.007	-0.008
**87.076**	0.002	0.013	**189.226**	0.009	0.003	**260.233**	-0.002	-0.054
**87.082**	0.007	-0.049	**189.241**	0.010	-0.023	**261.052**	-0.005	-0.038

**87.087**	0.015	-0.016	**190.077**	-0.050	0.045	**261.075**	0.041	0.040
**87.092**	-0.015	0.002	**190.082**	0.027	-0.033	**261.085**	0.020	0.013
**87.098**	-0.015	0.007	**190.088**	0.006	-0.015	**261.097**	0.016	0.014
**87.103**	-0.024	0.005	**190.185**	0.012	-0.014	**261.107**	0.023	0.052
**87.109**	0.009	-0.006	**190.193**	-0.055	0.008	**261.113**	0.017	0.048
**87.115**	0.016	0.013	**190.209**	-0.011	-0.053	**261.122**	0.002	-0.038
**87.126**	0.028	0.036	**190.217**	-0.005	-0.011	**261.129**	0.008	0.022
**87.131**	0.017	0.027	**190.229**	-0.065	0.014	**261.135**	0.013	-0.013
**87.136**	0.025	0.010	**190.242**	0.027	0.008	**261.141**	0.017	0.019
**87.142**	0.091	0.074	**190.25**	0.011	0.004	**261.15**	0.007	0.007
**87.147**	0.011	0.016	**190.258**	-0.003	-0.022	**261.16**	0.038	0.040
**87.156**	0.043	0.057	**190.266**	0.008	0.050	**261.173**	0.009	0.011
**87.161**	0.028	0.004	**190.278**	-0.036	0.051	**261.188**	0.034	0.086
**87.169**	0.033	0.037	**190.297**	0.007	-0.004	**261.198**	0.013	-0.002
**87.18**	0.022	0.031	**190.306**	0.007	0.010	**261.207**	0.001	-0.036
**87.191**	0.007	0.010	**190.314**	-0.012	0.003	**261.245**	0.003	-0.029
**88.027**	0.030	0.057	**191.083**	-0.161	0.053	**261.254**	-0.005	-0.038
**88.033**	0.032	-0.009	**191.1**	0.089	0.045	**262.027**	-0.001	-0.050
**88.063**	0.126	0.039	**191.107**	0.077	-0.099	**262.043**	0.007	-0.022
**88.068**	-0.024	0.000	**191.113**	0.016	-0.022	**262.06**	0.012	0.024
**88.09**	0.087	0.073	**191.122**	0.029	0.085	**262.069**	0.034	0.049
**88.095**	0.037	-0.079	**191.22**	0.006	0.006	**262.075**	0.002	-0.030
**88.101**	0.001	-0.007	**191.228**	0.074	0.037	**262.086**	0.013	0.005
**88.117**	-0.015	0.003	**191.236**	-0.003	-0.007	**262.099**	0.008	-0.025
**88.123**	-0.012	0.005	**191.252**	-0.005	-0.021	**262.11**	0.019	0.015
**88.139**	0.002	-0.003	**191.26**	0.075	-0.009	**262.118**	0.012	0.024
**88.145**	0.070	0.076	**191.268**	0.023	-0.025	**262.126**	0.027	0.029
**88.15**	0.054	-0.036	**191.276**	0.010	-0.012	**262.136**	0.033	0.040
**88.156**	0.026	0.003	**191.285**	0.005	-0.005	**262.145**	0.051	0.075
**88.164**	0.044	-0.005	**191.293**	0.040	0.008	**262.153**	0.004	-0.064
**88.169**	0.053	-0.038	**191.3**	0.040	-0.022	**262.158**	0.006	-0.011
**88.175**	0.043	-0.016	**191.309**	0.062	-0.037	**262.164**	0.052	0.058
**88.18**	0.020	0.005	**191.316**	0.019	0.024	**262.173**	0.008	-0.065
**88.194**	0.092	-0.004	**191.325**	0.009	-0.010	**262.183**	0.007	0.007
**88.205**	0.005	-0.005	**192.02**	0.010	0.010	**262.201**	0.007	-0.005
**88.214**	0.009	-0.061	**192.043**	0.010	0.012	**262.211**	0.008	-0.006
**89.01**	0.034	0.031	**192.051**	0.010	0.006	**262.23**	-0.005	-0.038
**89.018**	0.043	0.037	**192.112**	-0.094	0.006	**263.066**	-0.002	-0.026
**89.049**	0.110	0.065	**192.119**	-0.007	-0.023	**263.099**	0.000	-0.017
**89.071**	-0.013	0.018	**192.184**	0.012	-0.007	**263.106**	0.015	0.031
**89.076**	0.075	0.104	**192.192**	0.005	-0.007	**263.114**	-0.066	-0.024
**89.087**	0.005	-0.009	**192.222**	0.013	0.004	**263.119**	0.007	0.032
**89.093**	-0.034	0.012	**192.229**	0.007	0.005	**263.125**	0.050	0.085
**89.098**	0.114	0.095	**192.258**	-0.006	-0.013	**263.13**	0.012	-0.043
**89.104**	0.014	-0.017	**192.265**	-0.109	-0.006	**263.135**	0.000	-0.012
**89.109**	0.005	-0.009	**192.273**	-0.013	0.000	**263.142**	0.007	-0.025
**89.115**	0.012	0.006	**192.282**	-0.014	0.000	**263.151**	-0.001	0.015

**89.12**	0.029	0.021	**192.287**	-0.005	-0.006	**263.16**	0.012	-0.011
**89.126**	0.056	0.070	**192.294**	-0.008	-0.004	**263.17**	0.002	0.016
**89.131**	-0.009	-0.059	**192.339**	-0.014	0.000	**263.179**	0.005	0.001
**89.137**	0.010	0.012	**192.347**	-0.017	-0.001	**263.189**	-0.026	-0.003
**89.142**	-0.003	-0.063	**193.028**	0.046	0.054	**263.217**	0.017	0.028
**89.148**	0.103	0.131	**193.034**	-0.007	-0.062	**263.226**	-0.014	0.004
**89.153**	0.000	-0.034	**193.049**	0.011	0.043	**263.304**	0.014	0.039
**89.159**	0.010	0.012	**193.054**	0.005	-0.005	**264.031**	0.012	0.017
**89.17**	0.068	0.069	**193.062**	0.007	0.005	**264.059**	0.024	0.029
**89.175**	-0.011	-0.053	**193.07**	0.014	0.041	**264.088**	-0.134	0.015
**90.041**	0.146	0.063	**193.078**	0.005	-0.027	**264.095**	-0.052	0.041
**90.046**	0.016	0.012	**193.09**	0.024	-0.002	**264.102**	0.022	-0.034
**90.076**	0.130	-0.024	**193.096**	0.046	0.054	**264.111**	0.033	0.039
**90.082**	-0.016	0.000	**193.104**	0.035	0.023	**264.122**	0.013	-0.012
**90.101**	0.018	-0.059	**193.111**	-0.113	-0.069	**264.127**	0.018	0.012
**90.106**	0.031	-0.027	**193.116**	-0.015	-0.006	**264.135**	0.018	0.004
**90.112**	0.012	0.035	**193.123**	0.024	0.031	**264.143**	-0.013	0.004
**90.129**	0.012	0.017	**193.152**	-0.012	0.005	**264.149**	0.019	0.054
**90.14**	0.006	-0.027	**193.16**	0.005	-0.009	**264.159**	0.004	0.017
**91.027**	-0.143	0.060	**193.188**	-0.022	0.005	**264.168**	0.004	0.028
**91.036**	0.009	0.059	**193.2**	0.012	0.035	**264.186**	0.019	0.018
**91.046**	-0.019	0.002	**193.209**	-0.002	-0.026	**264.202**	0.010	0.010
**91.055**	0.041	0.056	**193.225**	0.044	0.046	**265.051**	0.002	-0.030
**91.062**	0.014	0.044	**193.233**	0.007	-0.010	**265.065**	0.015	0.026
**91.07**	-0.014	0.004	**193.262**	0.010	0.003	**265.087**	0.013	-0.002
**91.082**	-0.021	0.001	**194.024**	0.029	0.030	**265.093**	0.001	0.027
**91.087**	0.015	0.022	**194.038**	0.010	0.010	**265.1**	-0.059	0.034
**91.104**	0.015	0.014	**194.054**	0.034	0.043	**265.106**	-0.002	-0.055
**91.11**	0.040	0.086	**194.063**	0.012	0.017	**265.112**	0.032	0.026
**91.132**	0.010	0.014	**194.068**	0.075	0.082	**265.121**	-0.015	0.026
**92.06**	0.049	0.019	**194.074**	0.018	0.021	**265.128**	0.017	-0.011
**93.037**	-0.133	0.020	**194.08**	-0.013	-0.043	**265.134**	0.003	-0.008
**93.042**	-0.102	0.017	**194.089**	-0.091	0.023	**265.141**	0.013	-0.002
**93.059**	0.013	-0.014	**194.096**	-0.087	0.007	**265.15**	-0.046	0.013
**93.065**	0.009	-0.005	**194.106**	0.009	-0.022	**265.159**	0.023	0.000
**93.07**	0.008	-0.004	**194.114**	0.047	0.040	**265.164**	0.016	0.048
**93.076**	0.040	0.034	**194.121**	0.079	-0.095	**265.178**	-0.025	0.004
**93.082**	0.000	-0.068	**194.129**	0.054	0.016	**265.188**	0.001	0.011
**93.087**	0.006	-0.034	**194.137**	0.010	0.003	**265.197**	0.026	0.006
**93.093**	-0.222	0.031	**194.163**	0.058	0.084	**265.206**	0.012	0.035
**93.104**	0.010	0.012	**194.17**	0.009	-0.051	**265.216**	-0.027	0.012
**93.109**	0.006	-0.002	**194.178**	0.010	0.006	**265.226**	-0.002	-0.018
**93.132**	-0.014	0.004	**194.195**	0.016	0.008	**265.235**	0.023	0.003
**93.138**	-0.112	0.007	**194.203**	0.006	-0.031	**265.255**	0.022	0.029
**93.155**	-0.121	0.022	**195.02**	0.097	0.020	**265.263**	-0.002	-0.026
**93.186**	-0.213	0.032	**195.026**	0.015	-0.007	**265.273**	0.020	-0.023
**93.223**	-0.205	0.032	**195.034**	-0.032	0.036	**266.073**	0.009	0.009

**93.248**	-0.231	0.035	**195.042**	-0.027	0.004	**266.08**	0.007	-0.022
**93.271**	-0.182	0.029	**195.047**	0.011	-0.007	**266.096**	0.015	0.011
**93.294**	-0.063	0.011	**195.056**	0.001	-0.001	**266.102**	0.010	0.012
**93.39**	-0.016	0.001	**195.065**	0.046	0.031	**266.114**	0.019	0.039
**93.412**	-0.016	0.001	**195.076**	-0.005	0.017	**266.123**	0.008	-0.016
**93.435**	-0.016	0.001	**195.085**	0.001	0.026	**266.134**	0.039	0.085
**93.574**	-0.112	0.014	**195.094**	0.010	0.012	**266.141**	-0.003	-0.044
**93.58**	-0.023	0.006	**195.113**	-0.019	-0.023	**266.146**	0.010	0.006
**93.591**	-0.095	0.018	**195.119**	-0.028	-0.009	**266.152**	0.058	0.026
**93.596**	-0.030	0.002	**195.126**	0.140	-0.021	**266.161**	0.060	0.016
**93.607**	-0.024	0.007	**195.133**	0.012	0.017	**266.169**	0.014	-0.007
**93.616**	-0.112	0.018	**195.159**	0.004	0.017	**266.189**	0.036	0.017
**93.63**	-0.024	0.007	**195.167**	-0.005	-0.049	**266.219**	0.009	0.009
**93.636**	-0.083	0.017	**195.2**	0.009	0.011	**266.228**	0.021	-0.048
**93.658**	-0.028	0.010	**195.208**	0.009	-0.012	**266.237**	0.007	0.005
**94.036**	0.009	0.003	**195.216**	0.102	-0.067	**266.262**	-0.002	-0.026
**94.044**	-0.201	0.033	**195.241**	0.004	-0.032	**267.095**	0.061	-0.027
**94.05**	0.031	0.040	**195.249**	0.066	-0.048	**267.101**	0.070	0.047
**94.07**	-0.031	0.020	**195.257**	0.012	0.017	**267.11**	-0.113	0.040
**94.095**	-0.201	0.062	**195.265**	0.010	0.010	**267.118**	-0.091	0.010
**94.101**	0.026	-0.017	**195.282**	0.021	-0.013	**267.125**	0.006	-0.011
**94.12**	-0.207	0.034	**196.018**	0.006	-0.002	**267.131**	0.003	-0.003
**94.135**	-0.012	0.003	**196.044**	0.005	-0.005	**267.137**	0.009	-0.010
**94.141**	-0.139	0.027	**196.055**	0.018	0.012	**267.147**	0.013	-0.028
**94.146**	-0.014	0.002	**196.06**	-0.012	0.003	**267.156**	0.009	0.004
**94.158**	-0.024	0.005	**196.066**	0.089	0.071	**267.165**	0.021	0.029
**94.163**	-0.139	0.027	**196.072**	0.038	0.008	**267.184**	0.002	-0.028
**94.186**	-0.041	0.006	**196.078**	0.007	-0.004	**267.194**	0.015	0.020
**94.195**	-0.048	0.003	**196.092**	0.014	-0.006	**267.204**	0.005	0.004
**95.032**	0.113	0.075	**196.101**	-0.002	-0.026	**267.246**	-0.012	0.003
**95.038**	-0.008	-0.006	**196.115**	-0.012	0.003	**268.023**	0.017	0.004
**95.043**	-0.205	0.033	**196.121**	0.124	0.069	**268.035**	0.009	-0.041
**95.052**	0.036	0.038	**196.127**	0.009	-0.014	**268.083**	0.020	0.009
**95.057**	0.013	-0.011	**196.158**	0.010	0.006	**268.09**	0.041	0.030
**95.064**	0.005	0.051	**197.037**	-0.005	-0.038	**268.095**	-0.012	0.005
**95.069**	0.067	0.018	**197.063**	0.013	-0.031	**268.103**	0.068	0.092
**95.074**	-0.008	0.007	**197.07**	0.003	-0.034	**268.111**	-0.031	0.034
**95.092**	-0.016	0.023	**197.077**	-0.037	0.011	**268.119**	-0.058	-0.021
**95.097**	0.005	0.043	**197.084**	0.065	0.110	**268.124**	-0.028	-0.050
**97.011**	0.087	0.034	**197.09**	-0.036	0.005	**268.13**	-0.042	0.002
**97.016**	-0.011	0.045	**197.095**	0.036	0.045	**268.139**	0.032	-0.025
**97.044**	0.109	0.073	**197.106**	0.025	-0.013	**268.145**	0.023	-0.026
**97.049**	0.005	-0.039	**197.131**	0.018	0.007	**268.152**	-0.008	0.017
**97.078**	0.038	0.061	**197.138**	-0.003	-0.002	**268.161**	0.029	0.045
**97.083**	0.058	-0.015	**197.144**	0.018	-0.018	**268.19**	0.026	0.044
**97.1**	0.010	-0.014	**197.152**	0.006	0.006	**268.2**	0.012	-0.020
**97.105**	-0.029	0.000	**197.169**	0.054	0.049	**268.237**	-0.004	-0.048

**97.112**	-0.010	-0.005	**197.176**	0.001	0.019	**268.282**	0.009	0.011
**97.123**	-0.004	-0.006	**197.184**	-0.001	-0.033	**268.325**	0.008	-0.004
**97.129**	0.014	0.034	**198.041**	0.010	0.014	**269.022**	0.017	0.018
**97.135**	0.006	0.006	**198.053**	0.002	-0.030	**269.041**	0.010	0.006
**98.049**	0.040	0.077	**198.06**	0.037	0.046	**269.094**	-0.013	0.001
**98.075**	-0.027	0.010	**198.069**	0.040	0.017	**269.099**	-0.159	0.018
**98.08**	0.116	0.027	**198.074**	0.031	0.032	**269.109**	0.113	0.147
**98.101**	0.010	-0.017	**198.084**	0.015	0.007	**269.119**	0.014	-0.066
**98.106**	0.051	0.002	**198.107**	0.021	-0.006	**269.127**	0.033	-0.052
**98.112**	0.014	0.007	**198.115**	0.009	-0.039	**269.132**	0.017	-0.027
**98.13**	-0.008	-0.008	**198.121**	0.019	-0.011	**269.14**	0.000	-0.007
**98.135**	0.170	0.070	**198.126**	-0.011	-0.053	**269.146**	-0.006	-0.002
**98.156**	0.093	0.034	**198.132**	0.098	0.095	**269.16**	-0.012	0.009
**98.161**	0.022	0.030	**198.139**	0.054	0.040	**269.169**	0.009	0.050
**98.176**	0.008	-0.004	**198.144**	-0.012	0.004	**269.179**	0.026	0.026
**98.182**	0.006	-0.011	**198.173**	0.010	0.006	**269.189**	-0.014	0.004
**98.187**	0.013	0.003	**198.18**	0.051	0.067	**269.208**	0.003	-0.012
**98.97**	0.058	-0.018	**199.044**	0.002	-0.030	**269.217**	0.111	0.083
**99.029**	0.081	0.018	**199.054**	0.036	0.039	**269.222**	0.010	-0.015
**99.065**	0.117	0.086	**199.062**	0.004	-0.029	**269.246**	-0.012	0.003
**99.07**	0.006	-0.007	**199.07**	-0.120	0.018	**269.255**	0.028	-0.012
**99.144**	0.018	-0.010	**199.077**	-0.042	0.002	**269.285**	0.016	0.018
**99.15**	-0.013	0.001	**199.098**	-0.013	0.001	**269.293**	0.039	0.044
**99.205**	0.010	0.014	**199.107**	-0.012	0.005	**269.323**	0.006	-0.002
**100.094**	0.125	-0.072	**199.115**	0.033	0.075	**269.332**	0.039	0.067
**100.179**	0.007	0.010	**199.121**	0.105	-0.066	**269.341**	0.020	0.014
**100.202**	-0.016	0.000	**199.162**	-0.016	-0.003	**269.377**	0.027	0.018
**100.208**	0.012	0.011	**199.195**	-0.016	0.001	**270.027**	0.020	0.034
**100.214**	-0.026	0.001	**199.204**	-0.012	0.005	**270.036**	0.030	-0.026
**100.24**	0.003	-0.008	**199.228**	0.003	-0.009	**270.045**	0.022	0.032
**100.269**	-0.028	0.010	**199.237**	0.015	-0.005	**270.074**	0.012	0.024
**100.379**	0.003	-0.009	**199.245**	0.003	-0.008	**270.084**	0.005	0.026
**100.391**	0.014	-0.018	**199.259**	0.003	-0.009	**270.09**	0.012	0.017
**100.398**	0.032	0.020	**199.27**	0.029	-0.026	**270.101**	0.010	0.006
**101.045**	0.154	0.094	**200.02**	0.009	0.003	**270.116**	0.038	0.034
**101.05**	-0.005	-0.038	**200.036**	0.010	0.014	**270.125**	0.031	-0.043
**101.087**	0.116	0.165	**200.043**	0.007	0.010	**270.132**	0.013	0.055
**101.093**	-0.034	-0.081	**200.05**	-0.003	-0.043	**270.138**	0.008	-0.030
**101.1**	0.058	-0.103	**200.056**	0.027	0.016	**270.145**	0.029	0.015
**101.124**	-0.012	0.005	**200.062**	0.048	-0.054	**270.15**	0.007	-0.005
**101.129**	0.010	0.021	**200.072**	0.029	0.026	**270.17**	0.007	-0.009
**101.148**	-0.012	0.005	**200.082**	0.002	0.019	**270.179**	0.013	-0.016
**101.153**	0.106	0.085	**200.092**	0.021	0.022	**271.072**	0.011	0.016
**101.171**	-0.012	0.003	**200.098**	0.017	0.032	**271.107**	0.006	0.022
**101.177**	0.056	0.017	**200.11**	0.000	0.008	**271.113**	-0.007	-0.003
**101.183**	-0.001	-0.001	**200.116**	0.037	-0.066	**271.121**	0.024	0.043
**101.195**	-0.012	0.003	**200.123**	0.038	-0.041	**271.128**	0.005	-0.017

**101.201**	0.039	-0.055	**200.133**	0.049	0.048	**271.134**	-0.010	0.021
**101.206**	0.041	-0.024	**200.139**	0.025	-0.008	**271.157**	-0.002	-0.068
**101.227**	0.016	-0.011	**200.151**	0.048	0.023	**271.162**	0.034	0.009
**102.04**	0.042	-0.005	**200.163**	-0.003	-0.001	**271.178**	0.004	0.030
**102.045**	0.006	-0.026	**200.188**	0.030	0.035	**271.187**	0.043	0.091
**102.074**	0.078	0.063	**200.196**	0.002	-0.003	**271.195**	0.065	-0.032
**102.103**	-0.021	0.003	**200.221**	0.107	0.128	**271.277**	0.025	0.013
**102.127**	0.012	0.017	**200.23**	0.009	0.011	**271.287**	0.018	0.010
**102.133**	0.002	-0.003	**200.254**	0.010	0.003	**271.307**	0.024	0.070
**102.15**	0.051	0.038	**201.064**	-0.012	0.001	**271.315**	0.020	-0.028
**102.156**	0.017	0.027	**201.07**	0.042	-0.037	**271.326**	0.012	0.017
**103.023**	0.098	0.082	**201.075**	-0.016	-0.004	**271.336**	-0.002	-0.026
**103.028**	-0.004	-0.004	**201.084**	0.008	0.033	**271.345**	0.025	0.073
**103.058**	0.122	0.046	**201.102**	0.009	-0.010	**271.354**	0.010	0.006
**103.121**	-0.012	0.005	**201.11**	0.026	0.027	**271.364**	0.016	0.019
**103.127**	0.008	-0.009	**201.117**	0.047	0.064	**271.373**	0.027	-0.019
**103.147**	0.035	-0.019	**201.127**	0.040	-0.005	**271.383**	0.023	0.049
**103.153**	0.020	0.029	**201.137**	0.004	0.017	**271.388**	0.010	0.006
**103.174**	0.061	0.063	**201.209**	0.006	0.006	**271.412**	-0.002	-0.026
**103.189**	-0.011	-0.053	**201.234**	0.022	0.049	**272.041**	0.045	-0.006
**103.213**	-0.011	-0.053	**201.242**	-0.011	-0.053	**272.048**	0.016	0.019
**104.023**	0.009	-0.047	**201.268**	0.006	-0.019	**272.098**	-0.001	0.016
**104.051**	0.163	0.024	**201.276**	0.041	0.049	**272.104**	-0.009	-0.067
**104.059**	-0.027	0.002	**201.284**	0.028	0.030	**272.114**	0.090	0.069
**104.082**	0.013	-0.043	**201.292**	0.010	0.003	**272.119**	0.019	0.019
**105.036**	0.129	0.101	**201.317**	0.007	0.005	**272.124**	0.017	0.026
**105.045**	-0.019	-0.006	**202.039**	0.089	0.034	**272.129**	0.003	0.015
**105.065**	0.017	0.025	**202.046**	0.010	-0.027	**272.137**	0.005	-0.019
**105.071**	0.011	0.034	**202.066**	0.046	0.058	**272.142**	0.008	-0.037
**105.077**	0.007	0.025	**202.078**	-0.227	0.029	**272.149**	0.009	-0.010
**105.082**	-0.012	0.005	**202.085**	0.017	0.009	**272.16**	0.023	-0.024
**105.113**	0.009	0.009	**202.091**	0.095	0.058	**272.165**	0.010	-0.010
**105.125**	0.072	0.081	**202.102**	0.014	-0.013	**272.179**	-0.013	0.001
**105.137**	0.009	0.009	**202.107**	-0.001	-0.088	**272.185**	0.047	0.059
**105.142**	0.010	0.010	**202.113**	0.012	-0.020	**272.195**	0.031	0.037
**105.149**	0.021	0.019	**202.118**	0.008	-0.015	**272.203**	-0.006	0.000
**106.019**	0.006	-0.060	**202.124**	0.043	0.052	**272.213**	0.006	-0.004
**106.025**	0.076	0.070	**202.136**	0.051	-0.004	**272.242**	0.003	-0.009
**106.03**	0.029	0.033	**202.157**	0.017	0.014	**272.252**	-0.002	-0.026
**106.036**	0.002	-0.030	**202.166**	-0.006	-0.059	**272.292**	0.037	0.036
**106.045**	0.006	-0.027	**202.173**	0.017	0.027	**272.3**	-0.005	-0.038
**106.064**	0.083	0.029	**202.19**	-0.028	0.004	**272.342**	0.002	-0.029
**106.069**	0.051	0.048	**202.207**	0.017	0.027	**272.383**	-0.005	-0.038
**106.112**	0.020	0.022	**202.224**	0.000	-0.019	**273.04**	0.010	0.014
**107.049**	0.078	0.110	**202.265**	-0.038	0.010	**273.047**	0.014	-0.054
**107.054**	0.025	-0.061	**202.282**	-0.012	0.004	**273.052**	0.012	0.035
**107.1**	0.013	-0.012	**202.29**	-0.060	0.005	**273.094**	0.012	0.024

**108.035**	0.010	0.003	**202.332**	-0.016	0.000	**273.102**	0.012	0.021
**108.044**	-0.035	0.008	**203.073**	0.028	0.022	**273.121**	0.006	0.015
**108.049**	-0.042	-0.050	**203.078**	-0.114	-0.104	**273.13**	0.022	0.016
**108.055**	0.000	-0.007	**203.084**	0.057	0.171	**273.137**	0.050	0.052
**108.061**	0.012	0.027	**203.095**	0.029	-0.027	**273.145**	-0.016	-0.030
**108.067**	0.025	0.027	**203.103**	0.030	-0.053	**273.157**	0.005	0.007
**108.073**	0.056	0.071	**203.116**	0.003	-0.008	**273.166**	0.026	0.011
**108.081**	-0.010	-0.101	**203.132**	0.011	-0.004	**273.171**	0.010	0.006
**108.086**	0.009	0.011	**203.141**	-0.014	-0.090	**273.181**	-0.009	-0.006
**108.111**	0.017	0.027	**203.149**	0.063	0.069	**273.21**	-0.012	0.004
**109.035**	-0.011	-0.053	**203.156**	-0.016	0.000	**273.224**	0.001	-0.011
**109.044**	0.100	0.081	**203.166**	0.006	-0.002	**273.229**	0.017	0.025
**109.052**	-0.003	-0.044	**203.182**	-0.016	0.002	**273.258**	0.015	0.015
**109.063**	-0.011	-0.053	**203.19**	-0.021	-0.007	**273.267**	0.004	-0.034
**109.068**	0.012	0.017	**203.198**	-0.015	0.002	**273.302**	0.009	0.011
**109.078**	0.140	0.156	**203.207**	-0.012	0.005	**274.059**	0.025	-0.009
**109.084**	-0.009	-0.104	**203.216**	-0.002	-0.007	**274.159**	0.032	0.050
**110.044**	0.008	-0.006	**203.224**	-0.025	0.004	**274.166**	0.034	-0.007
**110.058**	-0.218	0.029	**203.232**	-0.002	-0.026	**274.173**	0.004	-0.019
**110.064**	0.124	0.090	**203.241**	-0.016	0.000	**274.178**	0.045	-0.040
**110.071**	0.034	0.021	**203.249**	0.021	-0.032	**274.245**	0.001	-0.007
**110.114**	0.021	0.020	**203.257**	0.020	0.021	**274.276**	0.004	-0.060
**110.255**	-0.223	0.031	**203.265**	-0.002	-0.026	**274.285**	-0.005	-0.038
**111.019**	0.019	0.007	**203.282**	0.016	0.018	**274.314**	0.002	-0.030
**111.025**	0.122	0.077	**204.053**	0.061	0.055	**275.111**	0.007	-0.004
**111.059**	-0.093	0.022	**204.059**	0.023	-0.019	**275.126**	-0.030	0.002
**111.064**	-0.110	0.012	**204.065**	0.007	-0.020	**275.133**	-0.018	0.020
**111.093**	0.205	-0.003	**204.073**	-0.036	0.004	**275.141**	-0.061	0.019
**111.098**	0.021	-0.020	**204.082**	0.022	0.037	**275.148**	-0.020	-0.039
**111.126**	0.007	-0.012	**204.087**	-0.067	0.020	**275.156**	0.052	-0.017
**111.152**	-0.022	0.000	**204.093**	-0.011	0.062	**275.162**	0.073	0.051
**111.157**	-0.016	0.012	**204.099**	-0.013	0.002	**275.174**	0.013	0.034
**111.163**	-0.124	0.014	**204.104**	0.114	-0.013	**275.197**	-0.015	0.003
**111.176**	-0.048	0.012	**204.11**	0.022	-0.050	**276.059**	0.048	0.027
**111.185**	-0.151	0.031	**204.118**	-0.002	-0.026	**276.066**	0.012	0.024
**111.191**	-0.047	0.000	**204.126**	-0.027	0.005	**276.1**	-0.012	0.005
**111.213**	-0.075	0.016	**204.151**	0.026	0.017	**276.116**	-0.002	-0.026
**111.243**	-0.059	0.012	**204.181**	0.019	0.020	**276.122**	0.006	-0.033
**111.249**	-0.042	0.002	**204.218**	0.040	0.045	**276.134**	0.078	0.125
**112.052**	0.034	0.019	**204.243**	0.017	0.027	**276.143**	0.016	-0.059
**112.058**	0.150	0.114	**204.248**	0.019	0.023	**276.148**	-0.040	0.009
**112.063**	-0.181	0.022	**205.036**	0.006	0.006	**276.156**	0.065	0.040
**112.069**	-0.050	0.011	**205.061**	0.005	-0.005	**276.166**	0.013	-0.019
**112.076**	0.007	-0.005	**205.087**	0.021	0.036	**276.227**	0.010	0.012
**112.092**	0.173	0.055	**205.094**	0.067	0.028	**277.09**	0.010	0.012
**112.097**	-0.005	-0.038	**205.102**	0.004	-0.008	**277.103**	0.003	-0.009
**112.141**	-0.007	-0.002	**205.132**	0.014	-0.019	**277.115**	0.006	-0.031

**112.147**	0.007	0.015	**205.14**	-0.007	-0.004	**277.129**	0.007	-0.002
**112.153**	0.015	0.013	**205.148**	0.004	-0.008	**277.135**	0.039	0.018
**112.171**	0.009	0.003	**205.165**	-0.014	0.002	**277.143**	-0.062	-0.017
**113.041**	0.158	0.095	**205.173**	0.003	-0.003	**277.151**	0.054	0.088
**113.048**	-0.012	0.005	**205.223**	0.010	0.003	**277.156**	-0.007	-0.005
**113.072**	0.057	0.049	**205.249**	0.022	0.033	**277.161**	-0.012	0.005
**113.078**	0.056	0.009	**205.256**	0.010	0.003	**277.192**	0.014	0.010
**113.104**	-0.018	-0.013	**205.287**	0.053	0.029	**277.222**	-0.002	-0.026
**113.11**	0.083	0.027	**205.294**	0.022	0.023	**277.231**	0.012	0.017
**113.12**	-0.011	-0.003	**205.32**	0.007	0.005	**277.262**	-0.002	-0.026
**113.141**	-0.023	0.015	**206.065**	0.019	0.060	**277.27**	0.054	0.022
**113.147**	0.012	-0.017	**206.073**	0.025	0.026	**277.305**	0.037	0.037
**113.163**	-0.028	0.003	**206.079**	-0.028	0.034	**278.062**	-0.011	-0.053
**113.172**	0.050	0.036	**206.085**	-0.088	0.006	**278.114**	0.019	0.036
**113.191**	0.006	-0.002	**206.091**	0.036	-0.030	**278.122**	0.059	0.047
**113.197**	0.026	0.009	**206.096**	0.013	-0.021	**278.128**	0.010	0.010
**113.222**	0.031	-0.010	**206.106**	0.011	0.031	**278.134**	0.027	0.009
**113.228**	0.002	-0.025	**206.114**	0.013	-0.011	**278.14**	0.008	-0.006
**113.246**	0.011	0.016	**206.122**	0.020	0.008	**278.145**	-0.002	-0.077
**113.254**	0.006	0.006	**206.139**	-0.013	0.001	**278.151**	0.012	0.035
**114.031**	0.013	0.008	**206.172**	0.011	0.016	**278.157**	0.006	0.006
**114.071**	0.129	0.060	**206.211**	0.043	0.050	**278.173**	0.017	0.027
**114.127**	-0.001	-0.015	**207.045**	0.018	0.016	**278.178**	0.010	0.006
**114.14**	-0.012	0.003	**207.05**	0.009	0.003	**278.207**	0.009	0.009
**114.146**	0.027	-0.018	**207.101**	-0.024	0.035	**278.216**	0.006	-0.002
**114.152**	0.005	-0.009	**207.108**	0.099	0.010	**278.227**	0.019	0.006
**114.158**	-0.028	0.010	**207.115**	0.003	-0.009	**278.236**	0.010	0.014
**114.165**	-0.009	-0.004	**207.216**	-0.006	-0.006	**279.092**	0.097	0.043
**114.171**	0.021	-0.007	**207.229**	-0.016	0.001	**279.097**	-0.006	-0.031
**114.177**	0.008	-0.041	**207.25**	0.000	0.001	**279.146**	-0.012	-0.025
**114.19**	0.005	-0.005	**207.28**	-0.007	-0.001	**279.175**	-0.013	0.001
**114.199**	0.015	-0.045	**207.292**	0.010	0.014	**279.214**	-0.013	0.001
**114.227**	0.045	0.040	**208.053**	0.010	0.006	**279.234**	-0.029	0.004
**115.021**	0.046	0.050	**208.096**	0.025	0.065	**279.253**	0.001	-0.007
**115.056**	0.041	-0.084	**208.105**	0.014	0.144	**279.263**	0.009	-0.001
**115.062**	0.127	0.137	**208.112**	-0.003	-0.101	**279.273**	-0.026	0.041
**115.074**	0.040	-0.051	**208.124**	0.086	-0.116	**279.308**	-0.025	-0.005
**115.079**	-0.023	0.005	**208.133**	0.004	-0.008	**280.095**	0.074	0.081
**115.089**	-0.052	-0.093	**208.153**	-0.015	0.002	**280.101**	-0.059	-0.002
**115.143**	-0.025	-0.008	**208.195**	-0.002	-0.026	**280.106**	0.039	-0.022
**115.148**	0.131	0.062	**208.204**	0.003	-0.009	**280.117**	-0.001	-0.045
**115.168**	-0.027	-0.007	**208.221**	0.013	-0.021	**280.164**	-0.006	0.009
**115.174**	0.143	0.059	**208.229**	0.023	0.008	**280.174**	0.022	-0.004
**115.18**	-0.013	0.002	**208.242**	0.056	0.032	**280.179**	0.015	0.012
**115.193**	0.005	-0.009	**208.25**	0.067	-0.053	**280.199**	0.007	-0.024
**115.199**	0.007	-0.072	**208.263**	0.009	0.011	**280.222**	0.008	-0.012
**115.205**	0.012	0.004	**208.272**	0.011	-0.001	**281.109**	-0.046	-0.024

**115.224**	-0.015	-0.061	**208.28**	0.028	-0.048	**281.116**	0.002	0.043
**115.23**	0.009	0.009	**208.288**	0.043	-0.055	**281.124**	-0.010	0.022
**115.237**	0.012	0.002	**208.301**	0.003	-0.002	**281.129**	0.049	0.068
**115.25**	0.040	0.021	**208.314**	0.005	-0.009	**281.137**	0.037	0.008
**116.05**	0.020	-0.080	**208.322**	0.007	-0.004	**281.144**	0.006	-0.047
**116.084**	0.131	0.030	**209.063**	0.005	-0.047	**281.152**	0.025	0.010
**116.144**	0.021	0.027	**209.069**	-0.012	0.004	**281.158**	0.009	-0.010
**116.156**	-0.015	0.003	**209.082**	-0.041	-0.012	**281.164**	0.002	-0.003
**116.169**	0.024	0.039	**209.09**	0.059	0.157	**281.179**	-0.001	-0.016
**116.175**	-0.006	-0.014	**209.096**	0.023	-0.023	**281.243**	0.027	0.024
**116.181**	-0.008	-0.005	**209.104**	0.027	-0.077	**281.258**	0.018	0.006
**116.188**	-0.013	0.000	**209.11**	0.003	-0.008	**282.092**	0.041	0.033
**116.206**	-0.015	0.003	**209.119**	0.013	-0.022	**282.098**	-0.055	-0.050
**116.213**	-0.008	-0.005	**209.135**	0.021	0.004	**282.108**	-0.078	0.088
**116.242**	0.050	0.019	**209.143**	-0.016	-0.082	**282.113**	-0.006	0.049
**117.036**	0.075	0.168	**209.169**	-0.016	-0.026	**282.123**	-0.005	-0.038
**117.071**	0.112	0.097	**209.176**	0.010	-0.004	**282.129**	0.012	0.024
**117.076**	-0.003	-0.044	**209.21**	0.003	-0.028	**282.136**	0.011	0.016
**117.133**	-0.016	0.000	**209.249**	0.007	0.010	**282.158**	0.010	0.003
**117.156**	-0.002	-0.026	**209.995**	0.012	0.017	**282.167**	0.009	0.003
**117.162**	0.021	0.002	**210.006**	0.007	0.010	**282.181**	0.017	0.016
**117.167**	0.122	0.085	**210.012**	0.012	0.017	**282.187**	0.010	0.010
**117.177**	0.007	-0.005	**210.035**	0.010	0.003	**282.201**	0.025	0.037
**117.188**	0.030	-0.002	**210.041**	0.009	0.011	**282.216**	-0.005	-0.038
**117.193**	0.146	0.085	**210.051**	0.021	0.000	**283.089**	0.026	-0.017
**117.219**	0.086	0.081	**210.058**	0.024	0.003	**283.094**	0.014	0.027
**117.225**	0.006	0.006	**210.068**	0.040	0.045	**283.1**	0.003	-0.021
**117.237**	-0.011	-0.053	**210.077**	0.045	0.030	**283.106**	-0.006	0.014
**117.244**	0.012	0.017	**210.085**	0.008	0.003	**283.118**	-0.046	0.009
**118.098**	0.092	-0.072	**210.092**	-0.002	-0.026	**283.125**	-0.012	0.005
**118.184**	-0.016	0.000	**210.098**	0.009	-0.004	**283.132**	-0.017	0.076
**118.261**	0.007	-0.009	**210.104**	0.002	-0.030	**283.138**	0.018	0.034
**118.28**	-0.026	0.002	**210.11**	0.020	0.042	**283.148**	-0.011	-0.056
**118.386**	0.017	-0.008	**210.126**	0.021	0.011	**283.153**	0.042	0.041
**118.411**	0.092	-0.085	**210.143**	0.085	0.107	**283.159**	0.007	0.005
**118.416**	0.008	0.035	**210.151**	0.038	0.045	**283.172**	0.009	-0.042
**118.461**	0.094	-0.068	**210.16**	0.006	-0.001	**283.177**	0.009	0.002
**118.487**	0.052	-0.050	**210.177**	0.025	0.024	**283.186**	0.006	0.010
**118.672**	0.005	-0.007	**210.185**	0.019	-0.004	**283.195**	0.022	0.026
**118.825**	0.004	-0.008	**210.193**	-0.002	-0.026	**283.211**	-0.013	0.011
**118.875**	0.011	-0.021	**210.211**	0.009	0.011	**283.216**	-0.013	0.001
**119.01**	0.009	0.011	**210.224**	-0.002	-0.026	**283.221**	-0.015	0.010
**119.047**	-0.054	-0.013	**211.003**	0.019	0.021	**283.23**	0.022	0.043
**119.054**	-0.015	0.002	**211.019**	0.002	-0.030	**283.259**	-0.002	0.016
**119.099**	0.088	-0.057	**211.027**	0.006	0.006	**283.27**	-0.006	-0.048
**119.192**	-0.016	0.000	**211.04**	-0.012	0.003	**284.081**	0.012	0.024
**119.199**	-0.038	0.034	**211.053**	0.014	0.039	**284.1**	0.027	0.015

**119.205**	0.106	-0.051	**211.06**	0.012	0.004	**284.113**	0.046	0.073
**119.224**	-0.039	0.008	**211.068**	0.008	-0.017	**284.119**	0.006	-0.002
**119.23**	0.100	-0.051	**211.074**	0.003	0.011	**284.132**	0.005	-0.057
**119.25**	-0.015	0.003	**211.084**	0.015	-0.036	**284.143**	0.014	-0.030
**119.256**	0.116	-0.018	**211.091**	0.036	0.054	**284.152**	0.035	0.040
**119.275**	-0.016	0.001	**211.097**	-0.025	0.004	**284.16**	0.003	-0.009
**119.281**	-0.016	0.000	**211.102**	-0.068	0.100	**284.174**	0.043	0.058
**119.287**	0.044	-0.031	**211.112**	0.026	-0.054	**284.182**	0.016	-0.001
**119.294**	-0.021	0.000	**211.117**	0.017	-0.013	**284.192**	0.042	0.055
**119.31**	0.016	0.049	**211.137**	0.003	-0.009	**284.217**	0.007	0.010
**119.316**	-0.012	0.005	**211.146**	-0.004	-0.010	**284.227**	0.020	0.020
**119.339**	0.082	0.027	**211.154**	0.003	-0.002	**284.256**	-0.006	-0.059
**119.348**	0.015	-0.022	**211.163**	-0.014	0.002	**284.262**	0.010	0.006
**119.362**	0.007	0.005	**211.172**	0.012	0.017	**284.275**	0.012	0.017
**119.367**	0.051	-0.020	**211.189**	0.040	0.042	**284.305**	0.007	0.005
**119.397**	0.021	-0.030	**211.196**	0.004	0.006	**285.104**	0.015	0.004
**119.403**	0.021	0.017	**211.231**	0.046	0.016	**285.115**	0.029	0.039
**119.412**	0.081	-0.022	**211.239**	0.045	0.046	**285.124**	-0.011	-0.029
**119.422**	0.009	-0.054	**211.265**	0.038	0.036	**285.133**	-0.005	-0.029
**119.435**	0.006	-0.007	**211.274**	0.023	-0.047	**285.14**	0.023	0.014
**119.441**	0.031	0.007	**212.024**	-0.002	-0.026	**285.147**	0.005	0.031
**119.983**	0.024	-0.015	**212.029**	0.002	-0.030	**285.154**	0.025	-0.026
**119.992**	0.085	0.057	**212.044**	0.004	-0.034	**285.165**	-0.001	0.065
**120.041**	0.131	-0.077	**212.054**	0.013	0.005	**285.172**	0.005	0.042
**120.047**	0.042	0.078	**212.066**	0.006	-0.014	**285.18**	-0.010	0.010
**120.076**	0.025	-0.044	**212.077**	0.028	0.020	**285.188**	0.010	-0.023
**120.082**	0.007	0.005	**212.082**	0.036	0.024	**285.195**	0.031	-0.002
**120.093**	0.075	0.073	**212.091**	0.047	0.047	**285.204**	0.036	-0.028
**120.099**	0.006	-0.050	**212.1**	0.013	-0.032	**286.007**	0.006	-0.002
**120.104**	0.031	-0.049	**212.105**	-0.016	0.000	**286.111**	0.004	0.009
**120.156**	0.033	0.037	**212.114**	-0.015	0.007	**286.117**	0.032	0.031
**120.162**	0.024	-0.019	**212.134**	0.026	-0.030	**286.123**	0.000	0.009
**120.188**	0.001	-0.007	**212.143**	0.079	-0.025	**286.129**	0.045	0.039
**121.062**	0.078	-0.056	**212.151**	0.019	0.029	**286.136**	0.018	0.022
**121.253**	0.010	-0.015	**212.159**	-0.017	-0.053	**286.141**	0.003	-0.003
**121.279**	-0.011	-0.051	**212.19**	-0.002	-0.026	**286.148**	-0.003	0.011
**121.301**	0.020	-0.094	**213.027**	0.052	0.056	**286.156**	0.006	-0.019
**121.33**	-0.023	-0.047	**213.046**	0.009	0.011	**286.162**	0.019	-0.050
**121.335**	0.127	-0.012	**213.056**	0.012	0.024	**286.169**	0.011	-0.024
**121.343**	0.006	0.006	**213.065**	0.018	0.030	**286.175**	0.013	-0.011
**121.35**	0.019	-0.002	**213.082**	-0.199	0.026	**286.18**	0.019	-0.030
**121.362**	0.072	-0.075	**213.095**	0.020	0.016	**286.195**	0.010	0.006
**121.368**	-0.006	-0.003	**213.105**	0.005	-0.072	**286.204**	0.025	0.011
**121.482**	0.010	0.014	**213.115**	0.015	0.009	**286.214**	-0.013	0.001
**122.018**	0.012	0.017	**213.124**	0.051	0.025	**286.244**	0.020	0.041
**122.024**	0.006	0.032	**213.133**	0.089	0.111	**287.14**	0.020	0.012
**122.066**	0.086	-0.039	**213.142**	0.067	-0.095	**287.148**	0.086	0.007

**122.092**	-0.016	0.001	**213.15**	0.018	-0.015	**287.156**	0.015	-0.041
**122.15**	0.004	-0.008	**213.167**	0.023	0.008	**287.251**	-0.001	0.018
**122.157**	-0.013	0.001	**213.223**	0.010	0.008	**287.265**	0.003	-0.031
**122.163**	0.043	0.001	**213.253**	-0.013	0.000	**287.35**	0.012	0.024
**122.169**	0.036	0.030	**213.261**	-0.037	0.013	**288.041**	0.019	0.016
**122.176**	0.016	-0.004	**214.033**	0.018	0.018	**288.133**	0.041	-0.083
**122.189**	0.025	-0.002	**214.04**	0.106	0.069	**288.141**	0.059	0.144
**122.195**	0.022	0.047	**214.048**	-0.036	-0.003	**288.148**	-0.007	0.010
**123.02**	0.025	-0.011	**214.065**	-0.027	0.002	**288.157**	0.017	-0.071
**123.026**	0.047	0.004	**214.074**	-0.013	-0.004	**288.166**	-0.003	-0.010
**123.058**	0.010	0.003	**214.083**	-0.046	-0.027	**288.183**	0.007	-0.008
**123.093**	0.052	-0.020	**214.092**	-0.061	-0.003	**288.19**	0.011	-0.009
**123.181**	0.018	0.037	**214.105**	0.047	0.045	**288.214**	0.086	0.017
**123.207**	-0.016	-0.008	**214.113**	0.089	0.024	**288.22**	0.005	-0.014
**123.213**	0.022	0.006	**214.118**	0.020	-0.039	**288.229**	0.018	-0.038
**124.068**	0.036	0.023	**214.126**	0.031	-0.036	**288.249**	0.006	-0.018
**124.074**	0.070	-0.012	**214.143**	0.006	-0.007	**288.258**	0.003	-0.008
**124.083**	0.027	-0.016	**214.152**	-0.029	0.027	**288.269**	0.011	0.016
**124.092**	-0.016	-0.009	**214.16**	0.004	0.011	**288.288**	0.008	-0.017
**124.099**	0.010	-0.005	**214.168**	-0.024	0.006	**288.298**	0.028	-0.029
**124.124**	0.002	-0.030	**214.177**	0.013	-0.016	**288.308**	0.010	0.012
**125.033**	0.068	0.030	**214.185**	-0.024	0.006	**288.318**	0.009	0.003
**125.04**	-0.002	-0.026	**214.194**	-0.009	0.001	**288.327**	0.012	0.017
**125.063**	0.007	0.014	**214.202**	-0.026	0.011	**288.338**	-0.001	-0.037
**125.072**	0.127	0.043	**214.211**	0.007	-0.005	**289.134**	-0.003	-0.045
**125.079**	-0.002	-0.026	**214.229**	0.003	-0.002	**289.143**	0.037	0.027
**125.105**	-0.002	-0.014	**214.237**	0.026	0.028	**289.15**	0.056	0.002
**125.122**	-0.013	0.001	**214.246**	0.079	0.008	**289.158**	-0.045	0.024
**125.138**	0.021	0.045	**214.254**	0.006	0.019	**289.163**	0.023	0.036
**125.144**	0.006	0.006	**214.263**	0.010	0.010	**289.173**	0.003	-0.008
**126.027**	-0.011	-0.053	**214.28**	0.007	0.005	**289.214**	0.012	0.035
**126.034**	0.026	0.030	**214.285**	0.012	0.024	**289.234**	-0.013	0.001
**126.053**	-0.222	0.034	**215.04**	0.011	0.004	**289.254**	0.074	0.055
**126.067**	0.140	0.084	**215.048**	0.017	-0.005	**289.274**	0.010	0.010
**126.073**	0.007	-0.005	**215.055**	0.012	-0.034	**289.284**	0.007	0.010
**126.102**	-0.003	0.008	**215.096**	0.032	0.039	**289.294**	0.059	0.052
**126.109**	-0.009	-0.080	**215.105**	0.053	0.133	**289.299**	0.000	-0.051
**127.009**	0.010	-0.007	**215.113**	-0.002	-0.056	**289.334**	0.010	0.010
**127.015**	0.027	0.049	**215.121**	-0.007	-0.063	**289.342**	0.012	0.017
**127.02**	-0.005	-0.038	**215.146**	0.006	-0.007	**289.375**	0.007	0.005
**127.048**	0.051	0.065	**215.155**	-0.010	-0.003	**289.388**	0.009	0.009
**127.055**	0.012	0.009	**215.173**	0.005	-0.009	**290.056**	0.017	0.027
**127.085**	0.134	0.070	**215.18**	0.012	0.002	**290.066**	0.000	-0.035
**127.09**	-0.017	-0.004	**215.212**	0.003	-0.009	**290.141**	0.029	0.034
**127.117**	0.090	0.057	**215.22**	-0.005	-0.001	**290.146**	0.009	0.011
**127.124**	-0.016	-0.013	**215.25**	0.006	-0.002	**290.158**	0.027	0.026
**127.143**	0.003	-0.008	**216.029**	0.010	0.006	**290.167**	0.011	-0.048

**127.156**	-0.008	-0.006	**216.037**	-0.001	-0.044	**290.172**	0.045	0.044
**127.17**	-0.026	0.003	**216.046**	0.039	0.035	**290.181**	0.003	-0.017
**127.177**	-0.016	0.000	**216.052**	0.007	0.005	**290.19**	0.059	-0.075
**127.183**	0.021	-0.002	**216.06**	0.021	0.018	**290.196**	0.019	-0.029
**127.189**	0.026	0.029	**216.067**	0.010	0.006	**290.21**	0.012	0.017
**127.196**	-0.010	-0.002	**216.076**	0.011	-0.022	**290.241**	0.010	0.014
**127.21**	0.003	-0.008	**216.084**	0.011	0.016	**290.256**	0.003	-0.009
**127.216**	0.029	0.037	**216.09**	0.021	0.023	**290.296**	-0.002	-0.026
**127.25**	0.040	0.074	**216.095**	0.009	0.009	**291.15**	-0.014	0.004
**128.041**	0.000	-0.033	**216.106**	0.003	-0.051	**291.159**	0.056	-0.011
**128.047**	0.009	0.011	**216.111**	0.020	0.012	**291.168**	0.009	0.032
**128.082**	0.131	0.001	**216.118**	0.014	0.017	**291.176**	-0.001	0.039
**128.135**	-0.015	0.003	**216.125**	0.064	0.078	**291.181**	0.003	-0.018
**128.149**	-0.014	0.000	**216.136**	0.029	-0.062	**291.188**	0.028	-0.041
**128.155**	-0.025	0.001	**216.148**	0.038	0.056	**291.193**	0.031	-0.003
**128.162**	0.044	0.060	**216.157**	-0.015	0.002	**291.203**	0.007	-0.014
**128.169**	0.005	-0.009	**216.17**	0.034	0.006	**291.259**	-0.012	0.003
**128.182**	0.001	-0.016	**216.178**	0.010	0.003	**291.288**	0.002	-0.042
**128.189**	0.054	0.025	**216.204**	0.022	0.012	**291.344**	0.012	0.017
**128.195**	0.005	-0.009	**216.217**	-0.005	-0.038	**292.064**	-0.002	-0.026
**128.202**	0.010	0.014	**217.054**	0.003	-0.014	**292.122**	0.009	0.003
**128.219**	0.009	0.004	**217.06**	0.009	-0.008	**292.139**	0.036	0.009
**129.03**	-0.015	-0.005	**217.067**	0.018	-0.008	**292.147**	0.044	0.059
**129.039**	-0.013	0.002	**217.126**	0.106	-0.073	**292.152**	-0.002	-0.026
**129.06**	-0.026	0.002	**217.261**	-0.068	0.007	**292.157**	0.042	0.026
**129.066**	0.108	0.112	**217.273**	0.130	-0.041	**292.163**	0.013	0.006
**129.072**	-0.017	-0.001	**217.283**	-0.015	0.003	**292.17**	0.012	-0.008
**129.081**	0.034	0.054	**217.29**	0.003	-0.009	**292.178**	0.015	-0.012
**129.088**	-0.025	-0.064	**217.298**	-0.066	0.012	**293.135**	0.007	0.005
**129.095**	0.037	-0.062	**217.307**	0.103	-0.045	**293.146**	0.011	0.001
**129.101**	0.108	0.091	**217.334**	-0.048	0.009	**293.155**	0.007	0.063
**129.158**	-0.014	0.004	**217.342**	0.133	-0.080	**293.161**	-0.002	0.030
**129.164**	0.002	-0.003	**217.377**	-0.011	-0.004	**293.211**	0.008	0.003
**129.191**	0.044	0.049	**217.385**	0.111	-0.084	**293.221**	0.007	0.012
**129.198**	0.008	-0.004	**217.393**	0.009	0.009	**293.231**	-0.013	0.001
**129.225**	0.129	0.092	**217.411**	0.016	-0.019	**293.266**	0.018	0.023
**129.231**	0.006	-0.002	**217.416**	0.039	-0.006	**294.065**	0.003	-0.009
**129.245**	0.023	0.023	**217.452**	0.035	-0.044	**294.078**	0.008	-0.015
**130.063**	0.171	0.049	**217.459**	0.005	-0.009	**294.098**	0.046	0.055
**130.068**	-0.009	-0.013	**217.486**	0.025	-0.025	**294.105**	0.017	0.013
**130.08**	-0.015	0.003	**217.493**	0.025	-0.031	**294.113**	0.018	0.030
**130.092**	0.047	0.034	**217.498**	0.013	-0.018	**294.122**	-0.005	-0.078
**130.098**	0.062	-0.016	**217.532**	0.016	-0.022	**294.13**	0.061	0.094
**130.104**	0.019	-0.022	**217.546**	0.038	-0.044	**294.149**	0.002	-0.027
**130.125**	0.007	-0.008	**217.551**	0.020	0.007	**294.159**	0.010	0.010
**130.132**	0.005	-0.005	**217.558**	0.014	-0.018	**294.175**	0.010	-0.008
**130.138**	0.008	-0.028	**217.567**	0.021	-0.029	**294.184**	0.017	0.012

**130.158**	0.020	0.012	**217.584**	0.020	-0.025	**294.198**	0.028	0.042
**130.164**	0.028	0.009	**217.592**	0.006	-0.011	**294.205**	0.019	0.028
**131.066**	0.088	0.157	**217.6**	0.005	-0.009	**294.214**	0.010	0.012
**131.072**	-0.021	-0.092	**217.623**	0.006	-0.011	**295.116**	0.010	0.012
**131.136**	0.002	-0.003	**218.027**	0.020	0.018	**295.125**	-0.015	0.009
**131.142**	-0.006	-0.071	**218.035**	0.014	-0.009	**295.133**	0.034	0.001
**131.149**	0.020	0.035	**218.045**	0.007	-0.004	**295.14**	-0.007	0.033
**131.162**	0.010	-0.006	**218.053**	0.020	-0.015	**295.147**	-0.002	-0.050
**131.169**	-0.016	-0.054	**218.062**	-0.008	0.010	**295.152**	0.039	0.046
**131.176**	0.010	0.037	**218.073**	-0.041	0.009	**295.199**	-0.015	0.003
**131.182**	0.010	-0.013	**218.081**	-0.016	0.000	**295.23**	-0.011	-0.053
**131.189**	0.002	-0.003	**218.119**	0.014	0.028	**295.24**	-0.005	-0.038
**131.196**	-0.029	0.003	**218.126**	0.074	-0.015	**295.261**	0.012	0.017
**131.209**	0.012	0.017	**218.134**	0.033	-0.057	**296.11**	0.013	0.015
**131.253**	0.010	0.010	**219.042**	-0.012	0.005	**296.119**	0.083	0.037
**132.04**	0.039	0.013	**219.052**	0.003	-0.009	**296.129**	0.032	-0.042
**132.067**	0.022	0.000	**219.061**	0.005	-0.005	**296.137**	0.000	0.025
**132.072**	0.107	0.029	**219.072**	0.055	0.017	**296.143**	0.006	-0.007
**132.078**	0.001	0.002	**219.078**	0.018	0.027	**296.177**	0.007	0.010
**132.142**	0.012	0.017	**219.09**	0.007	-0.011	**296.182**	-0.002	-0.026
**133.031**	0.014	0.039	**219.103**	-0.007	0.018	**296.222**	0.012	0.017
**133.058**	0.114	0.075	**219.115**	-0.011	0.056	**297.008**	-0.012	0.005
**133.065**	0.007	-0.028	**219.125**	0.075	-0.048	**297.014**	0.019	-0.007
**133.074**	0.021	-0.031	**219.133**	0.038	0.032	**297.076**	0.000	-0.049
**133.08**	-0.016	0.018	**219.141**	0.009	0.003	**297.084**	-0.075	-0.003
**133.087**	0.015	0.015	**219.15**	-0.012	0.004	**297.091**	-0.143	0.053
**133.093**	0.015	0.027	**219.194**	0.005	0.016	**297.096**	0.029	0.048
**133.1**	-0.014	0.002	**219.202**	-0.004	-0.014	**297.103**	0.038	0.017
**133.127**	-0.014	0.002	**219.21**	0.012	-0.005	**297.113**	0.041	0.022
**133.147**	-0.012	0.003	**219.219**	-0.012	0.005	**297.136**	0.012	0.017
**133.154**	0.016	0.012	**219.228**	0.007	-0.009	**297.155**	0.003	-0.009
**133.161**	0.006	0.006	**219.237**	-0.011	-0.053	**297.167**	-0.033	0.011
**133.167**	0.033	-0.037	**219.246**	0.023	0.059	**297.176**	-0.012	-0.023
**133.174**	0.011	-0.014	**219.258**	0.007	-0.009	**297.186**	-0.017	0.050
**133.181**	0.012	0.017	**219.271**	-0.011	-0.053	**297.195**	0.050	0.035
**133.198**	0.022	0.037	**219.288**	0.004	-0.027	**297.2**	-0.025	0.022
**134.048**	0.102	0.070	**220.041**	0.009	0.003	**297.206**	0.006	0.005
**134.055**	0.038	0.033	**220.048**	0.104	0.089	**297.215**	0.019	0.022
**134.072**	0.043	0.006	**220.053**	-0.006	-0.038	**297.235**	0.039	0.054
**134.079**	-0.039	-0.002	**220.058**	0.017	0.017	**297.245**	0.002	0.019
**134.085**	0.059	-0.039	**220.067**	0.007	0.010	**297.255**	0.012	0.024
**134.092**	0.056	0.055	**220.079**	0.017	0.027	**297.266**	0.009	0.011
**134.121**	0.015	0.011	**220.087**	0.010	0.003	**297.276**	0.054	0.029
**134.149**	0.026	0.023	**220.126**	-0.133	0.014	**297.286**	0.007	0.029
**134.156**	0.010	0.010	**220.136**	0.087	-0.054	**297.295**	0.012	0.024
**134.163**	0.021	0.018	**220.235**	-0.013	0.000	**298.086**	-0.015	0.002
**134.169**	0.024	0.039	**220.252**	0.010	0.003	**298.092**	-0.067	-0.026

**134.196**	0.014	0.039	**220.27**	0.014	-0.034	**298.1**	-0.027	0.004
**135.07**	0.086	-0.015	**220.278**	0.013	0.008	**298.112**	0.010	0.010
**135.075**	0.007	-0.009	**220.287**	0.044	-0.009	**298.123**	0.032	0.036
**135.134**	-0.023	-0.003	**220.3**	0.070	-0.063	**298.128**	0.019	0.012
**135.161**	-0.008	-0.005	**220.305**	0.010	0.006	**298.134**	0.021	0.030
**135.181**	0.010	0.012	**220.313**	0.017	0.007	**298.143**	-0.011	-0.053
**135.993**	0.132	0.042	**220.322**	0.047	0.077	**298.158**	0.019	0.022
**136.027**	-0.012	0.003	**220.331**	0.029	0.020	**298.176**	0.002	-0.030
**136.041**	-0.016	0.001	**220.339**	0.010	-0.032	**298.185**	-0.005	-0.038
**136.062**	0.029	0.056	**220.347**	0.011	0.016	**298.245**	0.010	0.014
**136.068**	0.019	0.076	**220.382**	0.005	-0.009	**299.054**	0.019	0.003
**136.075**	0.017	0.027	**220.387**	0.003	-0.002	**299.07**	-0.004	-0.041
**136.089**	-0.010	-0.026	**221.085**	-0.014	0.002	**299.088**	-0.013	0.001
**136.094**	-0.010	0.001	**221.094**	0.068	0.047	**299.1**	0.011	0.032
**136.103**	0.144	-0.034	**221.101**	0.010	0.051	**299.138**	-0.002	-0.026
**136.109**	0.005	-0.001	**221.112**	-0.006	0.019	**299.183**	-0.005	-0.038
**136.123**	-0.016	0.001	**221.119**	0.003	-0.031	**299.196**	-0.020	-0.042
**136.13**	0.003	0.026	**221.125**	0.018	-0.022	**299.205**	0.019	0.053
**136.15**	-0.016	0.001	**221.134**	0.021	-0.044	**299.213**	0.057	0.089
**136.157**	0.002	0.027	**221.14**	0.050	-0.014	**299.222**	0.056	-0.024
**136.171**	-0.012	0.005	**221.148**	-0.003	-0.009	**299.309**	0.021	0.038
**136.184**	0.043	0.084	**221.156**	-0.069	0.014	**300.065**	0.007	0.010
**136.191**	0.007	-0.009	**221.166**	-0.017	-0.001	**300.106**	0.019	0.021
**136.198**	0.007	-0.005	**221.192**	-0.046	0.003	**300.115**	0.003	-0.009
**136.212**	0.128	0.081	**221.218**	-0.013	0.001	**300.122**	0.010	0.012
**136.218**	0.037	-0.062	**221.227**	0.001	-0.045	**300.13**	0.026	0.008
**136.239**	0.027	0.005	**221.235**	0.004	0.004	**300.138**	0.007	0.010
**137**	0.050	0.058	**221.244**	-0.006	-0.026	**300.166**	-0.002	-0.044
**137.007**	-0.002	-0.050	**221.262**	0.051	0.017	**300.175**	0.020	-0.001
**137.022**	0.088	0.062	**221.27**	-0.002	-0.080	**300.186**	0.011	-0.005
**137.027**	-0.023	-0.003	**221.297**	0.009	0.003	**300.191**	0.020	0.033
**137.033**	0.069	0.048	**221.304**	-0.011	-0.053	**300.197**	0.017	0.018
**137.041**	0.004	-0.008	**222.055**	0.011	0.016	**300.206**	0.022	0.032
**137.061**	-0.027	-0.082	**222.061**	0.015	0.035	**300.213**	0.046	0.007
**137.067**	0.116	0.098	**222.069**	0.008	-0.004	**300.235**	0.019	0.028
**137.09**	0.000	-0.012	**222.074**	-0.012	0.004	**300.251**	0.002	-0.030
**137.096**	-0.008	-0.034	**222.082**	-0.006	-0.031	**301.112**	0.009	0.003
**137.101**	0.106	0.085	**222.09**	0.037	0.057	**301.141**	0.006	-0.061
**137.107**	0.006	-0.022	**222.097**	0.033	-0.025	**301.149**	0.035	0.052
**137.171**	0.004	-0.024	**222.105**	0.017	0.067	**301.154**	0.015	0.007
**137.984**	0.010	-0.044	**222.112**	-0.013	0.017	**301.16**	0.014	0.051
**137.991**	0.069	0.098	**222.118**	0.003	-0.003	**301.165**	0.034	0.039
**137.997**	0.034	0.024	**222.134**	0.001	-0.009	**301.172**	0.003	-0.002
**138.04**	-0.016	0.001	**222.141**	0.015	-0.011	**301.179**	-0.010	-0.004
**138.083**	0.085	-0.095	**222.148**	0.010	-0.020	**301.191**	0.009	0.003
**138.194**	0.012	0.045	**222.155**	0.065	0.061	**301.256**	0.017	0.027
**138.2**	0.007	0.010	**222.164**	-0.077	-0.040	**302.141**	0.007	0.005

**138.225**	-0.016	0.001	**222.261**	-0.011	-0.053	**302.148**	0.010	0.006
**138.26**	0.006	0.006	**222.277**	-0.005	-0.038	**302.156**	0.007	0.010
**138.393**	0.004	-0.014	**223.042**	0.191	-0.016	**302.162**	0.016	0.029
**138.407**	0.007	-0.045	**223.048**	-0.045	0.009	**302.169**	0.056	-0.051
**138.438**	0.013	-0.017	**223.066**	-0.048	0.014	**302.174**	0.033	0.064
**139.012**	0.034	0.032	**223.074**	-0.062	0.001	**302.184**	-0.040	-0.004
**139.045**	0.037	0.026	**223.086**	-0.015	0.022	**302.197**	0.010	-0.014
**139.083**	0.070	-0.051	**223.095**	0.046	0.049	**302.234**	0.012	0.035
**139.117**	0.020	0.028	**223.103**	0.020	0.029	**302.254**	0.010	0.014
**139.145**	-0.027	0.002	**223.112**	0.013	-0.062	**302.259**	0.010	0.012
**139.151**	-0.015	0.007	**223.12**	0.003	-0.008	**302.268**	0.012	0.017
**139.172**	0.019	0.010	**223.138**	-0.013	0.002	**302.274**	0.014	-0.018
**139.179**	-0.028	0.007	**223.147**	0.002	-0.003	**302.311**	0.017	0.027
**139.186**	-0.025	0.009	**223.155**	0.007	-0.016	**303.121**	-0.011	-0.001
**139.2**	0.046	-0.005	**223.19**	0.004	-0.024	**303.138**	0.030	0.043
**139.207**	0.103	0.004	**223.225**	-0.012	-0.007	**303.148**	0.073	0.047
**139.214**	-0.014	0.004	**224.045**	0.005	-0.036	**303.154**	-0.035	-0.024
**139.222**	0.010	-0.023	**224.051**	0.028	-0.032	**303.164**	-0.043	0.003
**139.228**	0.033	0.018	**224.059**	0.092	0.075	**303.17**	0.014	-0.014
**139.234**	0.059	-0.041	**224.064**	0.048	0.067	**303.175**	0.007	-0.004
**139.256**	0.029	-0.017	**224.073**	-0.026	0.002	**303.181**	0.007	-0.005
**139.262**	0.078	-0.013	**224.078**	-0.066	0.007	**303.262**	0.010	0.012
**140.048**	0.017	0.027	**224.085**	-0.047	0.014	**304.127**	-0.012	0.005
**140.069**	0.010	0.006	**224.093**	-0.026	-0.080	**304.14**	-0.002	-0.026
**140.08**	0.078	0.122	**224.101**	0.002	-0.017	**304.147**	0.041	0.051
**140.086**	0.053	-0.129	**224.111**	-0.019	0.004	**304.158**	0.043	-0.058
**140.092**	0.005	-0.014	**224.119**	0.044	0.020	**304.164**	0.021	0.071
**140.12**	0.117	0.102	**224.127**	0.078	0.037	**304.17**	0.028	0.028
**140.127**	0.004	-0.008	**224.137**	0.035	0.016	**304.179**	0.076	-0.086
**141.02**	0.018	0.023	**224.145**	0.006	-0.002	**304.231**	0.003	-0.009
**141.027**	0.003	-0.058	**224.154**	0.005	-0.005	**304.269**	0.012	0.024
**141.055**	-0.013	0.000	**224.159**	-0.040	0.004	**305.125**	0.022	0.020
**141.096**	0.046	-0.021	**224.176**	-0.030	0.004	**305.13**	0.018	-0.025
**141.162**	-0.015	0.002	**224.188**	0.005	-0.005	**305.138**	0.004	-0.038
**141.19**	-0.004	-0.024	**224.198**	0.013	-0.016	**305.144**	0.015	0.026
**141.197**	-0.057	0.003	**224.215**	0.022	0.037	**305.155**	0.004	0.064
**141.204**	0.012	0.027	**224.224**	0.019	0.029	**305.162**	0.017	-0.011
**141.211**	0.006	0.006	**224.232**	0.019	-0.010	**305.172**	0.029	-0.036
**141.218**	0.030	-0.035	**224.241**	-0.010	-0.070	**305.177**	0.007	-0.009
**141.225**	-0.030	-0.052	**224.25**	0.017	0.027	**305.186**	0.006	-0.007
**141.232**	-0.024	0.018	**224.259**	0.021	0.035	**305.213**	0.012	0.035
**141.238**	0.006	0.006	**224.268**	0.008	-0.040	**305.234**	0.002	-0.030
**141.266**	-0.020	0.020	**224.303**	0.009	0.003	**306.079**	-0.012	0.003
**142.051**	0.011	-0.030	**225.003**	0.043	0.026	**306.115**	-0.013	0.001
**142.059**	0.098	0.098	**225.014**	0.009	0.009	**306.125**	-0.012	0.004
**142.065**	-0.012	0.004	**225.07**	0.023	0.035	**306.132**	-0.007	0.014
**142.093**	0.014	-0.067	**225.075**	0.011	0.013	**306.14**	0.021	0.008

**142.099**	0.055	0.044	**225.085**	-0.051	0.002	**306.146**	0.017	0.010
**142.124**	-0.006	0.007	**225.093**	-0.003	0.027	**306.153**	0.027	0.008
**142.13**	0.003	0.017	**225.099**	-0.048	-0.011	**306.163**	0.040	0.008
**142.158**	0.005	-0.009	**225.104**	0.069	0.025	**306.176**	0.015	0.029
**142.187**	0.002	-0.003	**225.113**	0.013	-0.019	**306.182**	-0.007	-0.009
**143.009**	0.012	0.017	**225.12**	0.030	0.035	**306.196**	0.006	-0.007
**143.075**	0.146	0.111	**225.129**	0.013	0.001	**306.227**	0.006	-0.002
**143.08**	-0.011	-0.014	**225.137**	-0.022	0.005	**306.233**	0.009	0.009
**143.109**	0.023	0.022	**225.145**	0.009	-0.009	**307.123**	0.003	0.012
**143.148**	-0.012	0.005	**225.155**	0.017	0.000	**307.132**	0.020	0.030
**143.179**	0.006	-0.002	**225.163**	-0.009	0.025	**307.139**	0.017	-0.002
**143.207**	0.035	0.037	**225.172**	-0.015	-0.054	**307.145**	-0.027	0.004
**143.214**	0.107	0.078	**225.181**	0.008	-0.004	**307.153**	0.045	-0.025
**143.221**	0.009	0.009	**225.19**	-0.009	0.009	**307.16**	0.018	0.024
**143.235**	0.012	-0.003	**225.198**	-0.005	0.001	**307.168**	0.020	0.040
**143.243**	0.046	0.076	**225.233**	0.003	-0.009	**307.191**	-0.035	0.012
**143.264**	0.050	0.040	**225.26**	0.017	0.027	**307.262**	0.009	0.011
**143.271**	0.039	0.040	**226.048**	0.010	0.003	**307.273**	0.007	0.010
**143.299**	0.019	0.026	**226.053**	0.010	0.014	**308.034**	0.006	-0.002
**143.33**	0.010	0.012	**226.062**	0.062	0.038	**308.1**	0.012	0.035
**144.026**	0.002	-0.030	**226.07**	0.022	-0.005	**308.121**	-0.002	-0.026
**144.033**	-0.003	-0.039	**226.079**	0.014	-0.014	**308.138**	0.010	0.014
**144.039**	0.007	-0.018	**226.084**	0.026	0.062	**308.148**	0.031	-0.004
**144.075**	0.140	0.055	**226.095**	0.010	0.014	**308.154**	0.031	0.000
**144.081**	0.005	-0.009	**226.105**	0.010	0.003	**308.16**	0.059	0.088
**144.106**	0.069	0.061	**226.114**	0.023	0.022	**308.168**	0.004	-0.049
**144.112**	0.054	-0.018	**226.126**	0.034	0.005	**308.174**	0.008	-0.032
**144.134**	0.005	-0.009	**226.139**	0.021	0.023	**308.181**	0.049	-0.004
**144.141**	0.026	0.002	**226.157**	0.039	-0.054	**308.189**	0.006	-0.007
**144.148**	0.006	-0.011	**226.174**	0.012	0.017	**308.208**	-0.002	-0.026
**144.169**	0.020	0.009	**226.192**	-0.001	-0.069	**308.249**	-0.011	-0.053
**144.175**	0.021	0.013	**226.197**	0.041	0.012	**308.259**	0.010	0.003
**144.183**	0.021	0.021	**226.219**	0.010	0.014	**309.063**	0.010	0.012
**144.197**	0.017	0.015	**226.236**	0.010	0.010	**309.141**	0.018	-0.002
**144.208**	0.025	0.022	**227.044**	0.006	0.006	**309.152**	-0.005	0.021
**145.051**	0.023	0.008	**227.052**	0.022	0.023	**309.159**	0.000	0.001
**145.061**	-0.016	0.000	**227.06**	0.003	-0.009	**309.166**	0.007	0.061
**145.089**	0.119	0.138	**227.072**	-0.011	-0.053	**309.173**	0.001	0.014
**145.095**	-0.004	-0.121	**227.079**	0.007	0.053	**309.227**	0.017	0.027
**145.161**	-0.015	0.007	**227.085**	-0.064	-0.008	**309.268**	0.005	-0.027
**145.169**	0.018	-0.007	**227.091**	-0.072	0.012	**310.062**	0.006	-0.002
**145.176**	-0.008	-0.003	**227.097**	0.006	0.025	**310.067**	0.018	0.016
**145.183**	0.018	-0.008	**227.105**	0.041	0.032	**310.074**	-0.007	-0.069
**145.19**	0.023	0.023	**227.112**	0.022	0.015	**310.085**	0.020	0.025
**145.197**	0.051	0.005	**227.122**	0.028	0.023	**310.106**	0.007	-0.020
**145.204**	0.010	-0.008	**227.134**	-0.006	-0.043	**310.116**	0.035	0.033
**145.211**	0.034	0.012	**227.141**	0.020	0.025	**310.124**	0.006	0.006

**145.219**	0.009	-0.018	**227.149**	0.024	-0.028	**310.133**	0.025	0.030
**145.226**	0.030	-0.046	**227.158**	0.014	-0.066	**310.143**	0.027	0.044
**145.232**	0.029	0.019	**227.171**	0.029	0.065	**310.149**	-0.030	-0.042
**145.239**	0.120	0.124	**227.18**	0.003	0.006	**310.16**	0.000	0.015
**145.247**	0.006	-0.011	**227.255**	0.009	0.003	**310.176**	0.029	0.029
**145.257**	-0.002	-0.026	**228.007**	0.025	0.024	**311.073**	0.010	0.010
**146.05**	0.130	0.048	**228.012**	0.058	0.055	**311.092**	0.019	0.024
**146.059**	0.004	0.006	**228.02**	-0.011	-0.053	**311.097**	0.015	0.015
**146.075**	0.023	0.017	**228.026**	0.014	0.017	**311.107**	0.021	0.003
**146.084**	0.019	-0.044	**228.035**	0.007	-0.033	**311.117**	0.074	0.096
**146.09**	0.124	0.050	**228.045**	0.025	0.000	**311.127**	-0.017	-0.079
**146.116**	0.008	0.023	**228.057**	0.015	0.059	**311.135**	0.008	0.023
**146.122**	-0.007	-0.044	**228.079**	0.020	0.028	**311.156**	0.014	-0.026
**146.136**	0.003	-0.009	**228.084**	0.010	0.006	**311.166**	0.018	0.016
**146.144**	0.032	0.003	**228.092**	0.019	0.013	**311.186**	0.010	0.006
**146.151**	0.009	0.009	**228.097**	0.026	0.015	**311.206**	0.058	0.097
**146.158**	0.010	-0.022	**228.105**	0.007	-0.034	**311.222**	0.012	0.017
**146.173**	0.007	-0.005	**228.11**	0.018	0.042	**311.227**	0.010	0.006
**146.179**	0.009	0.009	**228.118**	0.026	0.028	**311.238**	0.003	-0.009
**147.042**	-0.026	0.002	**228.125**	0.001	-0.006	**311.248**	0.022	0.048
**147.057**	0.109	0.092	**228.134**	0.020	0.017	**311.258**	-0.011	-0.053
**147.063**	0.016	-0.008	**228.143**	0.032	-0.024	**312.116**	0.010	-0.053
**147.071**	-0.061	-0.002	**228.152**	0.014	-0.014	**312.127**	0.037	0.025
**147.081**	0.018	-0.014	**228.161**	-0.008	-0.031	**312.133**	0.014	-0.015
**147.087**	0.011	-0.021	**228.169**	0.028	0.061	**312.138**	0.028	0.036
**147.092**	0.003	-0.008	**228.178**	0.031	-0.030	**312.149**	0.012	0.017
**147.101**	-0.028	-0.004	**228.188**	0.006	-0.004	**312.155**	0.026	0.035
**147.108**	0.032	-0.048	**228.196**	-0.005	-0.038	**312.205**	0.009	0.009
**147.179**	0.007	0.005	**228.204**	0.010	0.014	**312.21**	0.010	0.014
**148.062**	0.086	0.011	**228.231**	0.014	0.039	**313.067**	0.029	0.025
**148.067**	0.009	0.025	**228.24**	0.009	0.009	**313.075**	0.043	0.053
**148.097**	0.007	-0.009	**229.043**	0.020	-0.033	**313.082**	0.011	0.004
**148.133**	0.029	-0.091	**229.048**	0.012	0.024	**313.096**	0.011	-0.084
**148.154**	0.012	0.017	**229.064**	-0.015	0.007	**313.104**	0.020	0.045
**148.161**	0.012	0.035	**229.07**	-0.013	0.001	**313.111**	0.007	0.010
**148.19**	0.016	0.030	**229.076**	0.011	0.012	**313.142**	-0.001	0.039
**148.211**	0.018	0.031	**229.083**	-0.003	0.006	**313.157**	0.012	0.024
**148.217**	0.012	0.035	**229.096**	0.009	0.025	**313.162**	0.010	0.003
**149.052**	-0.229	0.031	**229.101**	0.004	0.001	**313.167**	0.012	0.035
**149.074**	0.121	0.122	**229.109**	-0.026	-0.002	**313.173**	0.022	0.007
**149.08**	0.005	-0.040	**229.115**	0.001	-0.019	**313.179**	0.006	-0.002
**149.086**	0.084	-0.117	**229.121**	-0.012	0.005	**313.188**	0.021	0.052
**149.118**	0.008	0.008	**229.127**	0.015	-0.044	**313.194**	-0.011	-0.053
**149.189**	-0.018	0.021	**229.132**	0.007	-0.009	**313.203**	-0.007	-0.038
**149.196**	-0.074	-0.012	**229.137**	0.079	0.085	**313.213**	0.027	0.026
**149.225**	-0.015	-0.033	**229.144**	-0.040	-0.008	**313.22**	0.010	0.014
**150.039**	0.004	-0.008	**229.149**	0.021	0.020	**313.228**	0.009	0.003

**150.046**	0.024	0.044	**229.156**	0.025	0.020	**313.234**	0.019	-0.009
**150.054**	-0.172	0.022	**229.223**	0.006	-0.012	**314.11**	0.031	0.046
**150.065**	0.050	0.035	**229.241**	0.019	0.027	**314.117**	-0.015	0.007
**150.072**	0.037	0.012	**229.249**	0.019	0.018	**314.126**	0.028	0.032
**150.077**	-0.002	-0.044	**229.258**	0.007	0.005	**314.134**	0.010	0.014
**150.084**	0.008	-0.021	**229.271**	0.014	0.039	**314.139**	-0.002	-0.050
**150.089**	0.012	-0.017	**229.303**	0.007	0.005	**314.148**	0.002	-0.061
**150.095**	0.015	-0.021	**230.024**	0.021	0.024	**314.159**	0.003	-0.009
**150.106**	0.009	0.009	**230.03**	0.047	-0.039	**314.164**	0.017	0.024
**150.113**	-0.001	0.045	**230.036**	0.039	0.033	**314.171**	0.010	0.006
**150.141**	0.004	0.037	**230.072**	0.009	0.011	**314.211**	0.010	0.003
**150.148**	-0.010	-0.003	**230.081**	0.005	0.037	**314.232**	0.006	-0.024
**150.174**	0.011	0.016	**230.096**	0.021	0.027	**315.11**	0.012	-0.012
**151.066**	-0.022	0.130	**230.104**	0.071	-0.030	**315.116**	0.010	0.010
**151.075**	0.069	-0.109	**230.111**	0.022	0.036	**315.121**	0.021	0.021
**151.118**	0.003	-0.009	**230.128**	0.015	0.033	**315.129**	0.015	0.001
**151.148**	-0.002	-0.026	**230.134**	-0.002	-0.005	**315.142**	0.004	-0.036
**151.155**	-0.013	0.000	**230.139**	0.013	-0.014	**315.15**	0.033	0.044
**151.162**	0.002	0.006	**230.146**	0.026	0.039	**315.161**	0.026	-0.010
**151.169**	0.025	0.021	**230.155**	0.057	0.015	**315.169**	0.032	0.061
**151.177**	-0.002	-0.026	**230.164**	0.072	0.046	**315.175**	0.012	0.017
**151.184**	0.003	0.022	**230.19**	0.021	0.018	**315.186**	0.002	0.020
**151.191**	0.042	0.071	**230.199**	0.039	0.029	**315.192**	0.007	0.010
**151.198**	-0.003	-0.004	**230.235**	0.009	0.009	**315.202**	0.011	-0.022
**151.205**	-0.013	-0.077	**230.243**	0.007	0.005	**315.207**	0.006	-0.002
**151.22**	0.007	0.010	**230.279**	0.015	0.002	**315.212**	0.001	-0.038
**151.231**	0.007	-0.039	**231.054**	0.027	0.038	**316.096**	0.005	-0.020
**151.981**	0.006	0.006	**231.06**	0.050	-0.009	**316.114**	0.009	0.009
**152.045**	0.009	-0.017	**231.068**	0.007	0.010	**316.135**	0.010	0.014
**152.058**	-0.041	0.032	**231.082**	0.038	-0.005	**316.185**	0.025	-0.007
**152.067**	-0.012	0.005	**231.092**	-0.012	0.016	**316.191**	0.009	0.003
**152.084**	-0.060	0.065	**231.098**	0.002	0.016	**316.198**	0.030	0.076
**152.092**	0.080	-0.105	**231.107**	-0.021	-0.032	**316.208**	0.067	-0.101
**152.098**	0.021	0.000	**231.116**	0.014	0.013	**316.214**	0.032	-0.018
**152.157**	0.018	0.028	**231.121**	0.014	0.039	**316.221**	0.023	0.052
**152.183**	0.023	-0.034	**231.129**	0.029	-0.006	**316.29**	0.009	0.011
**152.193**	0.006	-0.011	**231.142**	0.011	-0.034	**316.3**	0.013	-0.010
**153.021**	0.030	0.051	**231.15**	-0.014	0.000	**316.31**	0.053	-0.010
**153.027**	-0.004	0.007	**231.209**	0.007	-0.004	**316.341**	0.003	-0.008
**153.044**	0.028	-0.027	**231.227**	-0.015	-0.085	**316.347**	0.029	0.008
**153.054**	0.141	0.109	**232.055**	0.018	0.019	**316.352**	0.007	-0.008
**153.061**	0.016	0.000	**232.062**	0.021	0.026	**317.102**	0.017	0.027
**153.072**	-0.013	0.001	**232.068**	0.027	0.046	**317.113**	0.011	-0.026
**153.078**	-0.144	0.043	**232.077**	0.006	-0.033	**317.122**	0.004	-0.024
**153.084**	-0.022	-0.038	**232.105**	0.010	0.014	**317.136**	0.012	0.024
**153.09**	0.127	0.032	**232.112**	0.005	0.000	**317.146**	0.012	0.017
**153.097**	0.075	-0.039	**232.124**	0.079	0.025	**317.158**	0.010	0.003

**153.126**	-0.013	0.001	**232.131**	0.042	-0.019	**317.167**	0.012	0.036
**153.155**	-0.013	0.001	**232.136**	0.021	-0.007	**317.178**	0.025	-0.054
**154.049**	0.020	0.012	**232.142**	0.008	-0.014	**317.19**	0.019	0.026
**154.056**	0.027	0.039	**232.171**	0.009	0.002	**317.198**	0.031	0.036
**154.064**	0.036	0.026	**233.053**	0.014	-0.026	**317.207**	0.062	-0.074
**154.072**	0.017	-0.002	**233.078**	0.010	0.012	**317.212**	0.043	-0.051
**154.078**	0.019	0.038	**233.096**	0.031	0.033	**317.223**	0.003	-0.027
**154.085**	0.049	-0.022	**233.106**	0.001	-0.041	**317.247**	0.012	0.017
**154.092**	0.057	-0.024	**233.115**	-0.163	0.043	**317.306**	0.007	0.010
**154.121**	0.012	0.017	**233.121**	-0.035	0.000	**318.102**	0.000	-0.036
**155.009**	-0.004	-0.067	**233.132**	0.024	0.000	**318.109**	0.006	-0.002
**155.028**	0.019	-0.005	**233.176**	0.007	0.005	**318.123**	0.016	0.023
**155.071**	0.100	0.033	**233.203**	0.012	0.017	**318.135**	0.076	0.056
**155.077**	-0.001	0.006	**233.212**	0.015	0.017	**318.141**	0.040	0.018
**155.09**	-0.023	0.006	**233.238**	0.012	0.017	**318.154**	0.002	-0.030
**155.107**	0.112	0.075	**233.247**	-0.004	0.016	**318.168**	0.014	0.039
**155.113**	0.012	-0.048	**233.256**	-0.006	0.008	**318.187**	0.009	0.009
**155.14**	-0.003	0.021	**233.266**	-0.018	0.006	**318.204**	0.006	-0.002
**155.169**	0.003	-0.008	**233.275**	-0.012	0.003	**318.209**	0.010	0.014
**155.176**	-0.024	0.006	**233.283**	-0.098	0.015	**318.218**	0.003	-0.028
**155.191**	-0.015	0.003	**233.292**	-0.049	0.005	**318.229**	0.009	0.009
**155.198**	-0.012	-0.017	**233.301**	-0.038	0.002	**318.24**	0.012	0.017
**155.205**	0.029	0.070	**233.311**	-0.012	0.003	**318.25**	0.008	-0.034
**155.212**	0.001	-0.007	**233.319**	-0.016	0.000	**319.122**	0.000	-0.036
**155.232**	-0.006	0.021	**234.021**	-0.009	-0.061	**319.129**	-0.005	-0.038
**155.239**	0.003	-0.002	**234.041**	0.012	0.017	**319.148**	-0.015	0.007
**155.264**	0.010	0.012	**234.056**	0.024	0.011	**319.153**	0.045	0.037
**156.072**	0.108	0.080	**234.062**	0.020	0.009	**319.16**	0.050	0.066
**156.079**	-0.019	0.015	**234.072**	0.030	0.015	**319.171**	0.005	0.018
**156.092**	0.020	-0.073	**234.127**	-0.016	0.001	**319.176**	0.018	0.030
**156.097**	0.003	-0.008	**234.133**	-0.013	0.000	**319.201**	-0.023	-0.085
**156.104**	0.026	-0.013	**234.141**	-0.155	0.022	**319.243**	0.001	-0.039
**156.111**	0.039	0.031	**234.148**	0.123	-0.095	**320.111**	0.018	-0.002
**156.134**	0.002	0.013	**234.232**	-0.014	0.004	**320.117**	0.032	0.036
**156.14**	0.047	0.070	**234.241**	-0.015	0.002	**320.122**	0.022	0.033
**156.17**	0.010	0.010	**234.268**	-0.012	-0.028	**320.131**	0.047	0.041
**157.016**	0.011	0.016	**234.277**	-0.010	0.019	**320.141**	0.005	-0.034
**157.086**	0.075	-0.021	**234.285**	0.012	0.017	**320.151**	0.016	-0.017
**157.152**	-0.003	-0.013	**234.313**	0.010	-0.006	**320.186**	0.010	0.012
**157.159**	-0.017	-0.001	**234.318**	0.030	0.044	**320.197**	0.012	0.017
**157.181**	-0.026	0.000	**234.358**	0.011	-0.013	**320.207**	0.007	0.005
**157.188**	0.011	-0.003	**234.367**	0.007	-0.009	**320.218**	0.004	-0.027
**157.204**	-0.037	0.004	**234.376**	0.005	-0.014	**320.227**	0.010	0.014
**157.211**	-0.003	-0.034	**234.381**	0.025	-0.031	**320.238**	0.009	-0.029
**157.218**	0.048	0.030	**234.42**	0.007	-0.008	**320.248**	0.002	-0.030
**157.226**	-0.020	-0.001	**234.425**	0.008	-0.004	**321.113**	0.010	0.012
**157.233**	-0.038	0.005	**235.073**	-0.011	-0.053	**321.123**	0.047	0.019

**157.239**	0.012	-0.043	**235.09**	0.003	-0.003	**321.134**	0.016	-0.053
**157.247**	0.022	0.028	**235.101**	0.018	0.029	**321.142**	0.029	0.041
**157.269**	0.000	-0.009	**235.109**	0.090	0.090	**321.15**	0.019	0.016
**157.276**	0.040	0.011	**235.116**	-0.022	-0.050	**321.159**	0.006	0.020
**158.027**	0.012	0.024	**235.122**	-0.033	0.017	**321.173**	-0.011	0.014
**158.057**	0.012	0.024	**235.13**	-0.005	0.009	**321.182**	-0.043	0.006
**158.065**	0.028	0.037	**235.139**	0.010	-0.006	**321.193**	-0.018	0.005
**158.076**	0.081	0.042	**235.147**	0.093	-0.109	**321.203**	-0.002	0.035
**158.086**	-0.026	0.002	**235.236**	0.037	-0.010	**321.213**	0.017	0.027
**158.094**	0.037	0.024	**235.264**	0.007	-0.009	**321.224**	-0.005	-0.086
**158.1**	-0.005	-0.038	**235.269**	0.015	0.009	**321.235**	0.017	0.017
**158.107**	0.022	-0.069	**235.304**	0.032	-0.074	**321.246**	0.022	0.030
**158.115**	-0.009	-0.012	**235.336**	0.025	-0.020	**321.26**	0.012	0.017
**158.122**	0.011	-0.007	**235.373**	-0.005	-0.038	**322.125**	0.011	-0.010
**158.152**	-0.013	0.020	**236.034**	0.093	0.056	**322.134**	0.078	0.120
**158.158**	0.012	0.029	**236.04**	-0.003	-0.021	**322.14**	0.046	-0.042
**158.188**	0.034	0.046	**236.045**	0.021	0.030	**322.147**	0.015	0.002
**158.217**	0.012	0.024	**236.079**	0.013	0.014	**322.154**	0.012	0.007
**159.1**	0.051	-0.001	**236.092**	-0.002	-0.010	**322.21**	0.012	0.024
**159.199**	0.038	0.097	**236.099**	-0.041	0.007	**322.236**	0.019	0.018
**159.229**	0.014	-0.003	**236.107**	0.071	0.019	**322.242**	-0.011	-0.053
**159.236**	-0.017	-0.003	**236.115**	0.010	0.062	**323.118**	0.018	0.030
**159.243**	0.007	-0.004	**236.12**	-0.005	-0.019	**323.128**	0.030	0.060
**159.276**	0.034	-0.023	**236.128**	0.006	-0.017	**323.137**	0.005	-0.053
**160.032**	-0.011	-0.053	**236.136**	0.031	-0.038	**323.145**	0.036	0.011
**160.063**	0.011	0.013	**236.144**	0.014	-0.021	**323.15**	0.035	0.031
**160.093**	0.040	0.033	**236.154**	0.032	0.001	**323.157**	0.010	0.003
**160.099**	0.097	-0.015	**236.163**	-0.015	0.007	**323.164**	0.007	0.005
**160.104**	-0.037	-0.002	**236.171**	-0.020	0.005	**323.21**	0.014	0.039
**160.168**	0.007	-0.005	**236.199**	0.007	-0.008	**323.237**	0.010	0.014
**161.048**	0.009	-0.052	**236.204**	-0.006	-0.003	**323.252**	0.007	0.010
**161.055**	0.017	-0.019	**237.075**	0.002	0.001	**323.279**	0.010	0.014
**161.066**	0.027	0.031	**237.087**	0.010	-0.009	**324.127**	0.009	0.009
**161.072**	0.095	0.077	**237.107**	0.085	0.077	**324.145**	0.026	-0.027
**161.08**	-0.032	0.007	**237.113**	0.006	-0.013	**324.155**	0.033	0.029
**161.088**	-0.008	-0.038	**237.123**	-0.002	-0.006	**324.165**	0.068	0.065
**161.096**	0.007	-0.013	**237.133**	0.005	-0.009	**324.171**	0.003	-0.009
**161.103**	0.005	-0.005	**237.153**	0.004	-0.005	**324.201**	0.009	0.009
**161.117**	-0.003	-0.008	**237.163**	0.007	-0.008	**325.134**	0.028	0.050
**161.177**	0.010	0.014	**237.195**	-0.007	-0.001	**325.14**	-0.049	0.008
**162.031**	0.010	0.003	**237.235**	0.016	0.012	**325.146**	-0.063	0.107
**162.042**	0.113	0.099	**237.244**	0.018	0.027	**325.154**	-0.005	0.020
**162.049**	-0.013	0.005	**237.253**	0.018	0.030	**325.162**	-0.003	-0.065
**162.058**	0.027	0.026	**237.28**	0.007	0.005	**325.169**	-0.008	-0.042
**162.069**	-0.002	-0.018	**237.29**	0.040	0.034	**325.192**	-0.013	0.000
**162.078**	0.033	0.020	**237.298**	0.007	0.010	**325.203**	0.012	0.024
**162.085**	0.033	-0.042	**237.308**	0.006	-0.002	**325.225**	0.006	0.006

**162.093**	0.050	0.078	**237.329**	0.041	-0.010	**326.11**	0.009	0.009
**162.099**	0.004	-0.023	**237.334**	0.007	0.010	**326.116**	-0.011	-0.053
**162.108**	0.021	-0.036	**238.053**	0.003	-0.009	**326.134**	0.002	-0.030
**162.115**	0.038	-0.016	**238.067**	0.011	0.016	**326.139**	0.014	0.007
**162.145**	0.017	-0.006	**238.082**	-0.013	0.001	**326.148**	0.030	0.016
**162.176**	0.007	0.005	**238.09**	0.006	0.010	**326.155**	0.043	0.037
**163.03**	0.017	0.006	**238.098**	0.025	0.031	**326.161**	0.010	0.006
**163.037**	0.072	0.076	**238.111**	0.013	0.027	**326.175**	0.012	0.024
**163.042**	0.021	-0.026	**238.12**	0.040	-0.033	**326.181**	0.006	0.006
**163.054**	-0.014	0.002	**238.126**	0.012	0.047	**326.191**	0.020	0.003
**163.064**	-0.016	0.000	**238.132**	0.009	0.012	**326.214**	0.014	0.039
**163.088**	0.030	-0.020	**238.143**	0.041	-0.090	**326.228**	0.009	0.003
**163.093**	0.063	0.041	**238.15**	0.003	-0.008	**327.082**	0.018	0.012
**163.1**	-0.019	-0.005	**238.165**	-0.010	-0.001	**327.092**	0.042	0.048
**163.131**	-0.003	-0.005	**238.183**	0.012	0.035	**327.097**	0.044	0.042
**163.138**	-0.001	-0.011	**238.191**	0.017	-0.067	**327.102**	0.024	-0.017
**163.145**	-0.002	0.001	**238.227**	0.039	0.057	**327.112**	0.012	-0.002
**163.161**	-0.009	-0.007	**238.236**	0.005	-0.009	**327.123**	-0.018	-0.007
**163.168**	0.018	0.040	**238.255**	0.011	0.016	**327.171**	0.021	0.028
**163.175**	0.022	0.011	**238.264**	0.028	-0.016	**327.177**	0.009	0.009
**163.183**	0.005	-0.009	**238.273**	-0.001	-0.032	**327.182**	0.007	0.030
**163.198**	0.031	0.036	**238.301**	0.025	-0.031	**327.192**	0.017	-0.050
**163.205**	0.027	0.025	**238.332**	0.007	0.005	**327.202**	0.008	-0.007
**163.213**	0.029	0.026	**239.091**	0.038	-0.007	**327.212**	0.037	0.036
**163.22**	-0.011	-0.053	**239.096**	0.035	0.018	**327.222**	0.007	0.010
**163.228**	0.017	0.027	**239.103**	0.014	0.057	**327.235**	-0.004	-0.048
**163.236**	0.033	0.034	**239.112**	-0.026	0.015	**328.105**	-0.011	-0.053
**163.243**	0.024	-0.004	**239.119**	0.028	-0.033	**328.122**	0.010	0.012
**164.046**	0.019	0.015	**239.126**	0.017	-0.038	**328.133**	0.031	0.027
**164.052**	0.005	-0.042	**239.142**	0.004	-0.024	**328.149**	0.010	-0.020
**164.058**	0.082	0.101	**239.151**	0.020	0.024	**328.16**	0.016	-0.008
**164.064**	0.002	-0.003	**239.169**	0.007	-0.014	**328.168**	0.033	0.034
**164.072**	0.003	-0.009	**239.178**	0.007	0.012	**328.177**	0.028	0.050
**164.081**	0.061	0.032	**239.187**	0.031	0.037	**328.19**	0.003	-0.049
**164.09**	-0.013	-0.049	**239.214**	-0.004	-0.003	**328.195**	0.007	0.005
**164.097**	0.082	0.089	**239.223**	0.019	-0.001	**328.214**	0.016	0.011
**164.105**	-0.003	0.001	**239.269**	0.017	0.027	**328.22**	0.017	0.027
**164.113**	0.029	-0.051	**240.054**	0.049	0.067	**328.229**	0.010	0.006
**164.12**	-0.006	-0.014	**240.06**	0.062	0.022	**329.13**	0.032	0.041
**164.126**	0.028	-0.002	**240.067**	0.003	0.036	**329.136**	0.028	0.035
**164.134**	0.032	0.049	**240.073**	0.011	-0.053	**329.145**	-0.008	-0.036
**164.156**	0.018	0.009	**240.078**	-0.040	0.009	**329.153**	0.034	0.064
**164.165**	0.026	0.010	**240.085**	-0.034	0.009	**329.158**	-0.011	-0.022
**164.171**	0.017	0.027	**240.094**	0.019	0.013	**329.167**	-0.003	-0.045
**164.196**	0.010	0.012	**240.103**	0.019	0.019	**329.173**	0.006	-0.002
**165.041**	-0.012	0.004	**240.112**	0.016	-0.010	**329.183**	0.012	0.024
**165.048**	0.015	0.021	**240.121**	0.014	0.046	**329.192**	0.010	0.006

**165.055**	0.083	0.045	**240.13**	0.048	0.038	**329.208**	0.009	0.011
**165.061**	-0.059	0.015	**240.14**	-0.008	0.001	**329.223**	0.026	0.029
**165.066**	-0.022	-0.015	**240.148**	-0.008	-0.016	**329.234**	0.016	0.018
**165.074**	-0.021	0.003	**240.157**	0.020	0.024	**329.245**	0.012	0.017
**165.081**	-0.031	0.008	**240.166**	0.028	0.043	**330.174**	0.036	0.073
**165.088**	0.030	0.046	**240.174**	0.002	-0.003	**330.182**	0.074	0.078
**165.096**	0.028	0.027	**240.203**	0.021	0.023	**330.191**	0.007	-0.068
**165.107**	-0.028	0.000	**240.212**	0.018	0.013	**330.199**	0.004	-0.031
**165.115**	0.022	0.046	**240.22**	-0.008	-0.094	**330.249**	0.012	0.017
**165.126**	0.054	0.073	**240.239**	0.015	0.016	**331.142**	-0.005	-0.038
**165.133**	0.009	-0.014	**240.248**	0.014	0.008	**331.172**	-0.002	-0.026
**165.155**	0.021	0.047	**240.998**	-0.017	-0.001	**331.177**	0.092	0.059
**165.164**	0.023	-0.005	**241.053**	0.012	0.035	**331.186**	0.037	0.092
**165.197**	0.009	0.009	**241.067**	-0.217	0.031	**331.193**	0.009	0.003
**166.027**	0.008	-0.004	**241.074**	-0.016	0.000	**332.178**	0.008	-0.026
**166.038**	0.017	-0.014	**241.103**	0.096	-0.089	**332.185**	0.020	-0.016
**166.05**	-0.011	-0.053	**241.112**	0.050	-0.010	**332.2**	0.022	0.030
**166.062**	0.047	0.043	**241.117**	0.039	0.035	**332.205**	0.010	0.010
**166.069**	-0.212	0.028	**241.123**	0.006	-0.002	**333.173**	0.026	0.029
**166.086**	0.022	0.021	**241.13**	0.026	0.034	**333.186**	0.000	-0.083
**166.097**	0.081	0.091	**241.184**	0.007	-0.004	**333.191**	0.046	0.069
**166.105**	0.110	-0.156	**241.202**	0.006	-0.004	**333.198**	0.029	0.068
**166.192**	-0.064	0.016	**241.219**	0.007	-0.004	**333.205**	-0.005	0.002
**166.211**	0.006	-0.007	**241.252**	0.027	-0.006	**333.284**	0.009	0.003
**166.222**	-0.133	0.025	**241.283**	0.005	-0.009	**334.144**	0.012	0.017
**166.241**	0.004	-0.008	**241.289**	-0.073	-0.002	**334.191**	-0.005	-0.054
**166.257**	0.003	-0.009	**241.294**	-0.014	0.002	**334.201**	0.026	-0.007
**166.268**	-0.231	0.029	**241.302**	-0.088	0.007	**334.207**	0.036	0.033
**166.274**	-0.013	0.001	**241.31**	-0.013	0.002	**334.215**	0.078	-0.037
**166.294**	-0.027	0.007	**241.32**	0.007	0.010	**334.222**	0.037	-0.037
**166.306**	-0.013	0.001	**241.329**	-0.007	0.007	**334.231**	0.012	0.024
**167.019**	0.053	0.012	**241.357**	-0.050	0.005	**334.263**	0.012	0.017
**167.025**	0.052	-0.006	**241.365**	-0.060	0.011	**335.129**	0.012	0.017
**167.031**	-0.014	0.004	**241.406**	-0.030	0.004	**335.141**	0.017	-0.051
**167.055**	0.036	0.037	**241.411**	-0.065	0.010	**335.148**	0.039	0.007
**167.063**	0.010	-0.015	**241.428**	-0.013	0.000	**335.155**	0.020	0.035
**167.069**	-0.068	0.029	**241.467**	-0.012	0.005	**335.16**	0.016	0.007
**167.077**	-0.058	0.028	**242.045**	0.010	0.010	**335.166**	0.018	0.025
**167.087**	0.034	-0.006	**242.054**	0.026	0.044	**335.177**	0.061	-0.020
**167.097**	0.023	-0.039	**242.064**	-0.006	0.009	**335.186**	0.007	0.014
**167.103**	0.081	0.001	**242.071**	-0.199	0.041	**335.206**	-0.038	-0.003
**167.11**	0.003	-0.008	**242.076**	0.006	0.027	**335.216**	-0.030	-0.002
**167.134**	0.056	0.058	**242.085**	0.019	-0.019	**335.226**	-0.036	-0.040
**167.156**	-0.016	0.000	**242.094**	0.029	0.021	**335.232**	-0.004	-0.006
**167.163**	-0.005	0.009	**242.103**	0.028	-0.069	**335.238**	0.019	0.063
**167.171**	-0.003	-0.055	**242.112**	0.054	-0.007	**335.249**	0.014	-0.007
**167.179**	0.018	0.018	**242.118**	0.005	-0.009	**335.28**	0.010	0.010

**167.201**	-0.011	-0.053	**242.139**	0.041	0.049	**335.291**	0.028	0.028
**167.232**	-0.011	-0.053	**242.148**	0.030	0.023	**335.313**	0.009	0.011
**168.033**	0.009	0.011	**242.157**	-0.015	0.002	**336.118**	0.009	0.003
**168.041**	0.014	0.039	**242.166**	0.012	0.017	**336.135**	0.020	0.025
**168.048**	-0.004	-0.042	**242.176**	-0.013	0.001	**336.148**	0.006	-0.021
**168.064**	0.009	0.037	**242.185**	-0.007	0.051	**336.154**	0.006	-0.002
**168.069**	0.028	0.052	**242.208**	-0.013	0.001	**336.162**	0.019	-0.009
**168.075**	0.040	-0.006	**242.993**	0.009	0.009	**336.177**	-0.002	-0.026
**168.081**	0.028	0.046	**243**	0.002	-0.030	**337.145**	0.009	0.011
**168.088**	0.019	-0.102	**243.006**	0.010	0.010	**337.159**	0.035	-0.013
**168.1**	0.005	-0.016	**243.043**	0.017	0.027	**337.168**	0.040	0.025
**168.126**	0.010	0.006	**243.062**	0.007	0.010	**337.176**	0.083	-0.072
**168.141**	0.003	-0.042	**243.079**	-0.065	0.021	**337.181**	0.017	-0.012
**168.149**	0.015	0.019	**243.085**	-0.030	0.003	**337.186**	0.009	-0.014
**168.176**	-0.011	-0.053	**243.091**	-0.039	-0.001	**337.194**	0.010	0.006
**169.005**	0.010	0.010	**243.108**	-0.025	0.007	**337.224**	0.009	0.011
**169.074**	-0.119	0.009	**243.115**	-0.013	0.001	**337.246**	-0.011	-0.053
**169.082**	-0.007	-0.137	**243.123**	-0.036	0.005	**337.256**	0.020	0.026
**169.089**	0.170	0.207	**243.132**	-0.085	0.011	**337.267**	0.030	0.021
**169.122**	0.012	-0.006	**243.143**	-0.064	0.007	**337.288**	0.004	-0.032
**169.146**	-0.013	0.001	**243.15**	0.059	0.011	**337.31**	0.026	0.025
**169.153**	0.086	0.063	**243.159**	0.088	0.085	**337.331**	-0.002	-0.026
**169.16**	0.008	0.032	**243.168**	-0.013	-0.002	**338.126**	0.015	0.022
**169.168**	-0.015	0.003	**243.175**	-0.053	0.008	**338.136**	0.012	0.017
**169.183**	0.015	0.049	**243.191**	0.032	-0.045	**338.15**	0.022	-0.022
**169.191**	-0.022	-0.005	**243.199**	0.034	-0.044	**338.166**	0.028	-0.014
**169.199**	-0.015	0.003	**243.207**	0.006	-0.007	**338.182**	0.005	-0.009
**169.207**	0.006	-0.007	**243.216**	0.012	0.011	**338.203**	0.012	0.017
**169.214**	-0.028	-0.011	**243.252**	-0.007	0.000	**339.134**	0.012	0.017
**169.221**	0.002	-0.026	**243.257**	-0.012	0.003	**339.147**	0.031	0.002
**169.229**	-0.008	-0.007	**244.068**	0.021	0.030	**339.156**	0.032	0.035
**169.245**	-0.015	0.007	**244.075**	0.023	-0.001	**339.163**	0.013	-0.030
**169.251**	0.032	0.031	**244.082**	0.014	0.019	**339.168**	0.012	0.024
**169.993**	0.009	0.009	**244.103**	-0.004	-0.075	**339.177**	-0.005	-0.054
**170.001**	0.007	0.005	**244.108**	0.042	0.044	**339.184**	0.027	0.038
**170.054**	0.014	-0.077	**244.116**	0.016	0.086	**339.201**	0.010	0.006
**170.061**	0.103	0.100	**244.124**	-0.006	-0.019	**339.26**	-0.005	-0.038
**170.066**	0.019	0.075	**244.13**	0.035	0.020	**340.124**	0.018	-0.002
**170.071**	0.006	-0.011	**244.139**	0.015	0.011	**340.13**	0.063	0.042
**170.077**	-0.012	-0.018	**244.144**	0.001	-0.005	**340.14**	0.039	-0.003
**170.086**	0.024	-0.052	**244.151**	0.024	-0.031	**340.145**	-0.001	-0.050
**170.091**	0.000	-0.051	**244.157**	0.027	-0.038	**340.156**	0.019	0.022
**170.098**	-0.013	0.002	**244.162**	0.007	0.019	**340.187**	-0.002	-0.026
**170.107**	0.003	-0.008	**244.167**	0.002	-0.003	**340.2**	0.009	0.009
**170.114**	0.023	0.012	**244.175**	0.016	0.000	**341.137**	0.006	-0.002
**170.122**	0.024	0.017	**244.193**	-0.012	0.004	**341.145**	0.029	0.034
**170.129**	0.002	-0.003	**244.203**	0.041	0.030	**341.152**	0.012	-0.019

**170.152**	0.053	0.048	**244.212**	0.066	-0.036	**341.162**	0.018	-0.003
**170.16**	0.050	0.004	**244.221**	0.006	-0.002	**341.176**	0.001	-0.053
**170.19**	0.027	0.042	**244.239**	0.010	0.010	**341.181**	0.002	-0.030
**170.213**	0.012	0.017	**244.249**	0.026	-0.010	**341.189**	0.032	0.047
**170.221**	0.010	0.006	**244.259**	0.006	-0.002	**341.236**	0.014	0.001
**170.997**	-0.011	-0.053	**244.286**	0.010	0.014	**341.242**	0.032	-0.002
**171.014**	0.006	0.006	**244.295**	0.013	-0.011	**341.285**	0.010	0.010
**171.064**	0.031	0.064	**245.01**	0.063	0.068	**342.15**	0.018	0.016
**171.086**	-0.013	0.010	**245.015**	0.062	0.000	**342.164**	0.022	-0.009
**171.094**	0.051	-0.028	**245.023**	0.008	-0.008	**342.171**	0.004	-0.039
**171.1**	0.086	0.115	**245.055**	0.014	0.039	**342.179**	0.016	0.020
**171.107**	0.003	-0.021	**245.084**	0.018	0.000	**342.242**	0.012	0.017
**171.131**	-0.030	0.008	**245.094**	-0.003	0.003	**342.287**	-0.011	-0.053
**171.138**	0.013	0.019	**245.103**	0.002	0.039	**343.169**	0.022	0.014
**171.161**	-0.025	0.004	**245.11**	-0.021	-0.025	**343.185**	0.010	0.003
**171.169**	0.000	-0.015	**245.12**	0.013	-0.022	**343.193**	0.015	-0.005
**171.2**	0.007	-0.009	**245.128**	0.003	0.038	**343.209**	0.013	-0.051
**171.207**	0.031	0.039	**245.133**	0.004	0.003	**343.217**	0.022	0.026
**171.23**	0.010	0.006	**245.142**	0.028	0.049	**343.224**	-0.011	-0.053
**171.237**	0.018	0.017	**245.147**	0.021	0.034	**344.164**	0.010	0.014
**171.246**	0.013	0.013	**245.157**	0.039	-0.026	**344.181**	-0.004	-0.041
**172.063**	-0.021	0.002	**245.165**	0.021	0.018	**344.188**	0.006	-0.002
**172.068**	-0.086	0.013	**245.17**	-0.021	-0.002	**344.194**	0.010	-0.007
**172.078**	-0.067	0.029	**245.183**	0.026	-0.013	**344.207**	-0.005	-0.038
**172.086**	0.067	0.004	**245.188**	-0.013	0.000	**344.214**	0.017	0.027
**172.091**	0.067	-0.061	**245.201**	0.005	-0.011	**345.182**	0.016	0.013
**172.096**	0.062	-0.005	**245.22**	0.012	0.000	**345.228**	0.012	0.017
**172.103**	0.024	0.021	**245.238**	0.009	0.011	**346.184**	0.010	0.010
**172.109**	-0.015	0.002	**245.256**	0.019	0.005	**347.178**	0.012	0.017
**172.124**	0.001	0.027	**245.262**	0.026	0.016	**347.185**	0.047	0.031
**172.135**	-0.011	-0.050	**245.275**	0.009	0.011	**347.195**	0.011	-0.059
**172.143**	-0.014	0.000	**245.293**	0.007	0.010	**347.202**	0.016	0.018
**172.159**	0.018	0.021	**245.298**	0.041	0.037	**347.213**	0.009	0.003
**172.166**	0.004	0.000	**246.025**	0.023	0.023	**348.17**	0.012	0.017
**172.227**	0.007	0.005	**246.031**	0.009	0.011	**348.184**	0.016	-0.055
**173.072**	0.030	-0.004	**246.085**	0.051	0.061	**348.195**	0.010	0.014
**173.08**	-0.039	-0.018	**246.09**	-0.020	-0.086	**348.206**	0.007	0.010
**173.087**	-0.015	-0.006	**246.096**	0.091	0.089	**349.154**	0.002	-0.030
**173.095**	0.091	0.075	**246.104**	0.021	0.049	**349.166**	0.020	0.023
**173.101**	0.017	0.027	**246.109**	-0.007	-0.027	**349.173**	0.015	-0.039
**173.113**	0.022	-0.002	**246.115**	0.016	0.003	**349.181**	0.021	-0.007
**173.174**	-0.013	0.002	**246.126**	0.006	-0.013	**349.196**	-0.026	0.012
**173.181**	-0.012	0.003	**246.137**	0.008	0.015	**349.207**	-0.015	0.002
**173.202**	0.007	-0.005	**246.146**	0.015	0.013	**349.254**	0.013	-0.014
**173.213**	0.002	0.006	**246.157**	0.016	-0.009	**350.162**	0.010	0.012
**174.036**	0.012	0.017	**246.168**	0.030	0.014	**350.187**	0.006	-0.023
**174.059**	0.021	0.025	**246.183**	0.036	0.057	**350.204**	0.010	0.003

**174.064**	0.009	0.011	**246.188**	0.018	0.014	**351.173**	0.006	0.006
**174.089**	-0.014	0.002	**247.047**	-0.011	-0.053	**351.178**	0.009	0.009
**174.098**	0.097	0.064	**247.057**	0.044	0.064	**351.183**	0.007	0.010
**174.104**	0.059	-0.081	**247.064**	0.020	-0.002	**351.195**	-0.015	-0.083
**174.111**	0.001	-0.008	**247.07**	0.011	0.016	**351.2**	0.025	-0.013
**174.193**	0.014	-0.016	**247.076**	0.003	0.012	**351.206**	0.023	0.020
**174.2**	0.012	-0.017	**247.088**	-0.002	-0.006	**351.214**	-0.001	0.021
**175.091**	0.036	0.047	**247.093**	-0.003	0.022	**351.232**	0.012	0.017
**175.097**	-0.014	-0.018	**247.102**	-0.049	-0.049	**352.162**	0.007	0.010
**175.191**	-0.016	-0.031	**247.113**	-0.070	0.051	**352.187**	0.007	0.005
**175.222**	-0.002	-0.034	**247.122**	-0.010	-0.003	**353.142**	-0.005	-0.038
**175.229**	-0.011	0.005	**247.132**	0.030	0.007	**353.151**	0.008	-0.005
**175.249**	0.010	0.012	**247.14**	0.039	0.013	**353.165**	0.022	0.019
**176.022**	0.009	0.011	**247.147**	0.001	-0.018	**353.233**	-0.011	-0.053
**176.035**	0.002	-0.030	**247.152**	0.021	0.032	**353.249**	0.007	0.010
**176.122**	0.071	-0.059	**247.158**	0.060	0.076	**354.181**	0.018	0.013
**176.223**	-0.002	-0.007	**247.163**	0.023	0.018	**354.196**	0.036	-0.052
**176.254**	0.009	-0.016	**247.177**	-0.016	-0.051	**354.203**	0.021	-0.034
**176.262**	0.031	0.021	**247.204**	0.002	-0.030	**355.141**	0.007	0.005
**176.282**	0.043	-0.061	**247.214**	-0.014	0.004	**355.155**	0.010	0.014
**176.317**	0.073	-0.092	**247.222**	-0.012	0.003	**355.166**	0.004	-0.017
**176.324**	0.007	-0.008	**247.231**	0.020	0.043	**355.172**	-0.009	-0.060
**176.352**	0.013	-0.014	**247.241**	0.057	0.041	**355.194**	0.030	0.035
**176.361**	0.007	-0.008	**247.25**	0.009	0.009	**355.224**	0.012	0.017
**176.383**	0.006	-0.004	**247.268**	0.012	0.004	**355.229**	0.009	0.003
**176.419**	0.007	-0.008	**247.278**	0.024	0.028	**355.235**	0.021	0.048
**177.042**	0.013	0.023	**248.072**	0.010	0.010	**356.155**	0.012	0.017
**177.053**	-0.016	-0.005	**248.116**	-0.026	-0.037	**356.187**	-0.004	-0.044
**177.059**	0.017	0.027	**248.125**	-0.018	0.034	**357.205**	0.016	0.018
**177.071**	-0.048	0.011	**248.131**	-0.029	0.003	**357.219**	0.010	0.003
**177.076**	0.000	-0.036	**248.136**	0.053	0.031	**357.231**	0.010	0.014
**177.084**	0.019	0.044	**248.143**	0.053	-0.050	**357.239**	-0.005	-0.038
**177.092**	0.056	0.027	**248.15**	0.030	-0.007	**358.225**	0.017	0.027
**177.1**	0.028	0.049	**248.162**	0.025	-0.037	**360.174**	0.009	0.009
**177.107**	-0.068	0.010	**248.2**	0.030	-0.013	**360.215**	0.010	0.010
**177.118**	0.034	-0.105	**248.208**	-0.013	0.000	**361.165**	0.012	0.017
**177.126**	-0.002	-0.010	**248.236**	0.049	-0.021	**361.173**	-0.002	-0.026
**177.142**	0.001	0.005	**248.246**	0.044	-0.015	**361.209**	0.010	0.014
**177.172**	0.003	-0.003	**248.283**	0.012	0.017	**363.16**	0.012	0.017
**178.036**	0.029	-0.011	**249.056**	0.006	0.006	**363.19**	-0.011	-0.053
**178.052**	0.015	0.009	**249.108**	-0.027	-0.022	**363.207**	0.010	0.010
**178.06**	-0.011	-0.029	**249.113**	0.053	0.072	**363.214**	0.012	0.017
**178.065**	0.002	0.054	**249.12**	-0.001	0.047	**363.231**	-0.002	0.012
**178.075**	0.000	0.031	**249.13**	0.002	-0.029	**363.237**	0.012	0.017
**178.081**	0.041	0.041	**249.138**	0.015	-0.019	**365.167**	0.010	0.014
**178.087**	-0.001	-0.004	**249.16**	0.001	-0.007	**365.173**	0.006	-0.002
**178.092**	0.043	-0.023	**250.051**	-0.006	-0.061	**365.181**	0.007	0.010

**178.098**	0.026	-0.064	**250.062**	0.002	-0.030	**365.193**	-0.011	-0.053
**178.105**	-0.012	0.007	**250.068**	0.013	0.001	**365.209**	0.009	0.003
**178.117**	-0.015	0.007	**250.089**	0.029	0.038	**371.008**	0.023	0.040
**178.125**	0.002	-0.003	**250.098**	0.010	0.014	**371.015**	-0.108	0.050
**178.14**	0.017	0.022	**250.104**	0.023	0.007	**371.022**	0.004	-0.012
**179.026**	0.097	0.005	**250.113**	0.020	0.016	**371.148**	0.013	-0.009
**179.031**	0.006	-0.014	**250.121**	0.040	-0.070	**371.158**	0.038	-0.008
**179.042**	0.049	0.069	**250.126**	0.005	0.048	**371.165**	0.013	-0.020
**179.048**	-0.085	-0.007	**250.132**	-0.026	0.061	**371.193**	0.025	0.031
**179.056**	-0.022	-0.004	**250.137**	-0.059	0.012	**371.198**	0.008	0.006
**179.064**	0.007	-0.066	**250.145**	0.010	0.002	**371.204**	-0.048	-0.003
**179.072**	0.011	0.008	**250.165**	0.004	-0.008	**371.21**	-0.016	0.055
**179.08**	0.003	-0.008	**250.178**	0.018	0.000	**371.22**	0.044	-0.055
**179.088**	0.002	-0.014	**250.187**	0.018	0.013	**371.304**	-0.012	-0.005
**179.095**	-0.008	-0.057	**250.197**	0.016	0.010	**371.311**	0.004	-0.008
**179.103**	0.009	-0.009	**250.215**	0.007	-0.022	**372.012**	-0.048	0.070
**179.115**	-0.012	-0.002	**250.224**	0.007	0.010	**372.021**	-0.033	0.010
**179.142**	-0.022	-0.005	**251.028**	0.010	0.003	**372.152**	0.066	0.137
**179.166**	0.009	0.004	**251.092**	0.006	-0.004	**372.157**	0.007	0.010
**179.178**	0.015	-0.002	**251.098**	0.028	0.044	**372.175**	-0.024	-0.053
**180.035**	0.003	0.016	**251.103**	0.042	0.035	**372.185**	-0.019	0.019
**180.042**	0.014	-0.019	**251.108**	0.013	-0.005	**372.191**	0.020	0.019
**180.047**	0.069	0.058	**251.115**	-0.004	-0.013	**372.197**	-0.016	0.088
**180.052**	0.025	-0.056	**251.12**	-0.012	-0.004	**372.21**	0.010	0.003
**180.059**	0.039	0.072	**251.142**	-0.013	0.001	**372.225**	0.030	0.051
**180.064**	-0.012	0.004	**251.169**	-0.007	0.000	**372.231**	-0.039	0.006
**180.077**	-0.030	-0.005	**251.178**	0.011	-0.022	**372.238**	0.000	0.020
**180.086**	0.021	0.004	**251.188**	-0.014	0.002	**372.247**	-0.033	0.006
**180.092**	-0.014	0.004	**251.198**	-0.016	0.000	**372.253**	0.003	0.044
**180.101**	-0.031	0.037	**251.207**	0.016	0.051	**372.26**	-0.008	-0.004
**180.108**	0.081	0.005	**251.244**	0.014	0.039	**372.266**	0.010	0.003
**180.115**	0.062	-0.064	**251.249**	-0.015	0.007	**372.277**	-0.025	0.004
**180.123**	-0.014	0.002	**251.281**	0.008	-0.021	**372.282**	0.002	0.055
**180.151**	0.009	-0.014	**252.021**	0.005	-0.056	**372.31**	0.002	-0.037
**180.174**	0.005	-0.005	**252.026**	0.040	0.017	**372.322**	0.020	0.046
**180.186**	-0.001	-0.042	**252.034**	0.050	0.084	**372.328**	0.012	0.056
**181.042**	0.054	-0.013	**252.041**	0.010	0.010	**372.333**	-0.033	0.005
**181.05**	-0.002	-0.026	**252.076**	0.009	0.009	**372.373**	0.008	0.037
**181.068**	0.046	0.068	**252.09**	0.009	-0.003	**372.399**	0.010	0.003
**181.074**	0.022	0.011	**252.097**	0.054	0.028	**373.006**	-0.016	-0.014
**181.079**	-0.001	-0.034	**252.105**	0.040	0.035	**373.016**	-0.092	0.072
**181.087**	0.013	0.001	**252.114**	-0.021	0.018	**373.021**	0.039	0.014
**181.096**	0.008	0.034	**252.122**	0.003	-0.009	**373.102**	0.041	0.041
**181.107**	-0.002	-0.026	**252.129**	0.008	-0.015	**373.113**	-0.016	-0.015
**181.115**	0.048	-0.013	**252.137**	0.006	-0.011	**373.148**	0.007	-0.009
**181.122**	0.003	0.005	**252.145**	0.023	0.024	**373.158**	0.013	0.017
**181.153**	0.006	-0.038	**252.155**	-0.002	-0.012	**373.164**	0.010	0.003

**182.046**	0.009	0.009	**252.168**	0.006	-0.007	**373.193**	0.018	0.034
**182.055**	0.041	0.044	**252.182**	0.016	0.031	**373.198**	-0.013	-0.003
**182.064**	0.040	0.043	**252.216**	0.008	-0.003	**373.204**	0.025	0.026
**182.072**	0.015	-0.045	**252.271**	0.012	0.024	**373.215**	-0.083	-0.013
**182.077**	0.027	0.014	**252.304**	0.012	0.024	**373.22**	0.029	0.034
**182.083**	0.017	0.000	**253.039**	-0.012	-0.071	**373.226**	-0.017	-0.063
**182.094**	0.018	0.014	**253.106**	0.007	0.028	**373.231**	0.005	0.029
**182.101**	0.004	-0.033	**253.116**	0.072	0.058	**373.237**	0.007	0.005
**182.112**	0.019	0.016	**253.125**	0.017	-0.004	**373.243**	0.002	0.019
**182.117**	0.012	0.035	**253.131**	-0.020	-0.014	**373.249**	-0.014	-0.018
**182.125**	0.025	0.029	**253.141**	0.006	-0.016	**373.258**	0.000	0.015
**182.141**	0.010	0.014	**253.16**	-0.007	-0.037	**373.316**	-0.005	-0.038
**182.156**	-0.003	-0.048	**253.169**	0.008	0.042	**374.007**	-0.017	-0.003
**183.039**	0.069	-0.090	**253.187**	-0.017	-0.002	**374.013**	-0.040	0.052
**183.045**	0.026	-0.029	**253.197**	0.001	0.003	**374.02**	-0.075	0.065
**183.054**	0.028	0.010	**253.215**	-0.013	0.001	**374.03**	0.052	-0.014
**183.059**	0.023	-0.008	**253.295**	0.024	0.000	**374.037**	0.007	0.005
**183.065**	-0.021	0.014	**254.042**	0.024	0.024	**374.074**	-0.017	-0.001
**183.072**	-0.036	0.030	**254.052**	0.013	0.009	**374.086**	-0.015	0.007
**183.078**	-0.097	0.097	**254.089**	0.004	-0.004	**374.098**	0.018	0.016
**183.086**	-0.014	0.000	**254.105**	0.038	0.061	**374.212**	-0.005	-0.038
**183.091**	0.048	0.002	**254.114**	0.028	0.063	**378.177**	0.005	-0.007
**183.097**	0.040	-0.050	**254.122**	-0.008	-0.037	**383.193**	0.012	0.017
**183.106**	0.010	-0.026	**254.131**	0.020	-0.027	**388.032**	0.006	0.038
**183.118**	-0.016	0.022	**254.137**	0.021	-0.033	**388.037**	0.073	-0.006
**183.129**	0.067	0.002	**254.143**	0.002	-0.019	**388.042**	0.005	-0.026
**183.137**	0.024	-0.006	**254.151**	0.005	-0.009	**388.048**	0.011	-0.019
**183.153**	-0.027	0.003	**254.158**	-0.012	0.005	**388.123**	-0.013	0.002
**183.161**	0.001	-0.032	**254.176**	0.019	0.024	**388.135**	0.002	-0.030
**183.169**	0.002	-0.030	**254.185**	-0.011	0.000	**388.158**	0.007	-0.009
**183.177**	0.008	-0.004	**254.27**	0.023	0.027	**388.211**	-0.005	-0.038
**183.201**	0.007	0.010	**255.051**	0.012	0.024	**388.216**	0.008	-0.004
**184.031**	0.006	-0.011	**255.099**	0.057	0.010	**388.233**	0.019	-0.015
**184.039**	0.078	0.011	**255.104**	-0.026	0.037	**388.239**	0.029	-0.024
**184.047**	0.050	0.046	**255.11**	0.032	0.003	**388.246**	0.019	-0.092
**184.06**	0.021	0.015	**255.116**	-0.033	-0.029	**389.026**	-0.002	0.006
**184.074**	0.022	0.011	**255.122**	0.010	-0.012	**389.032**	0.032	-0.021
**184.086**	0.028	-0.063	**255.127**	0.010	0.003	**389.038**	0.016	-0.098
**184.094**	0.005	-0.004	**255.139**	0.019	0.020	**389.043**	0.057	0.101
**184.106**	0.017	0.026	**255.153**	0.017	0.013	**389.052**	-0.016	0.001
**184.114**	0.003	-0.042	**255.158**	0.016	0.005	**389.105**	0.009	0.009
**184.122**	0.059	0.010	**255.167**	0.001	-0.019	**389.117**	-0.002	-0.026
**184.13**	0.033	-0.012	**255.176**	-0.011	-0.012	**389.128**	0.015	-0.005
**184.138**	0.016	-0.009	**255.186**	0.013	0.006	**389.152**	0.043	0.013
**184.146**	0.060	0.045	**255.194**	-0.007	-0.010	**389.158**	0.027	-0.030
**184.153**	0.013	-0.012	**255.204**	-0.020	-0.041	**389.163**	-0.013	0.001
**184.161**	0.010	0.006	**255.214**	-0.013	0.001	**389.174**	0.033	0.044

**184.205**	0.012	0.017	**255.26**	0.103	0.102	**389.181**	0.007	-0.004
**185.039**	-0.005	-0.038	**255.28**	0.003	-0.009	**389.186**	0.010	-0.013
**185.057**	-0.247	0.035	**255.289**	0.010	0.010	**389.197**	0.034	-0.045
**185.074**	0.011	-0.023	**255.298**	0.110	0.127	**389.204**	0.019	0.023
**185.095**	0.125	0.040	**255.317**	0.003	-0.009	**389.209**	0.041	-0.018
**185.102**	0.054	0.047	**255.335**	0.073	0.057	**389.215**	0.019	0.023
**185.108**	0.058	-0.077	**255.374**	-0.002	-0.026	**389.22**	-0.007	-0.007
**185.14**	0.016	0.004	**256.025**	0.010	0.012	**389.225**	0.005	-0.009
**185.148**	0.044	0.026	**256.053**	0.042	0.044	**389.233**	0.013	-0.006
**185.171**	0.003	-0.003	**256.058**	0.002	-0.030	**389.244**	0.012	0.017
**185.211**	0.014	0.039	**256.066**	0.026	0.037	**389.256**	0.005	-0.005
**185.243**	0.015	0.013	**256.09**	0.003	-0.002	**390.025**	0.016	-0.033
**185.251**	0.010	0.010	**256.098**	0.016	0.022	**390.031**	0.068	0.032
**185.26**	-0.246	0.037	**256.105**	0.023	0.035	**390.04**	0.064	0.003
**185.312**	-0.149	0.024	**256.11**	0.008	-0.037	**390.047**	-0.005	-0.052
**185.384**	-0.087	0.006	**256.12**	-0.003	-0.010	**390.058**	0.010	0.014
**185.42**	-0.091	0.007	**256.132**	0.009	-0.026	**390.173**	0.030	0.038
**185.429**	-0.159	0.030	**256.138**	0.059	0.052	**390.181**	0.007	0.005
**185.458**	-0.016	0.001	**256.144**	-0.004	0.070	**390.187**	0.011	0.016
**185.504**	-0.016	0.001	**256.15**	-0.011	-0.003	**390.215**	0.010	0.003
**185.901**	-0.060	0.008	**256.159**	0.049	-0.028	**391.03**	0.007	-0.005
**185.909**	-0.012	0.004	**256.169**	-0.011	-0.032	**391.193**	0.117	0.034
**185.931**	-0.135	0.016	**256.253**	0.012	0.035	**391.198**	-0.038	-0.054
**185.937**	-0.080	0.009	**256.28**	0.012	0.017	**391.203**	0.023	0.005
**185.945**	-0.012	0.004	**257.061**	0.011	-0.005	**391.213**	-0.016	0.001
**185.97**	-0.015	0.003	**257.072**	-0.014	0.004	**391.279**	-0.006	-0.019
**185.978**	-0.012	0.004	**257.091**	-0.025	0.005	**391.314**	-0.016	0.001
**185.988**	-0.015	0.002	**257.098**	0.007	-0.004	**391.325**	0.034	0.020
**185.996**	-0.022	0.008	**257.104**	0.009	0.010	**391.337**	-0.001	-0.005
**186.005**	-0.029	0.008	**257.116**	0.004	0.038	**391.349**	0.005	-0.009
**186.045**	0.147	0.099	**257.125**	0.012	0.008	**391.36**	-0.016	0.001
**186.054**	0.003	-0.043	**257.131**	-0.031	0.002	**391.371**	0.039	0.044
**186.061**	-0.240	0.034	**257.14**	-0.032	-0.056	**391.383**	0.020	-0.019
**186.09**	0.019	-0.002	**257.149**	-0.007	0.029	**391.389**	0.010	0.010
**186.098**	0.116	0.089	**257.154**	0.043	0.015	**391.413**	-0.016	0.001
**186.105**	0.081	-0.077	**257.161**	0.064	0.036	**391.418**	0.074	0.025
**186.129**	-0.002	-0.026	**257.17**	0.023	0.013	**391.425**	0.028	-0.013
**186.146**	0.033	0.003	**257.181**	0.014	0.030	**391.43**	0.000	-0.035
**186.153**	-0.005	-0.063	**257.224**	0.005	-0.009	**391.464**	0.013	-0.006
**186.163**	-0.008	0.025	**257.266**	0.025	0.024	**392.185**	0.009	-0.006
**186.17**	0.075	0.086	**257.276**	0.010	0.003	**392.191**	0.096	0.002
**186.177**	0.052	-0.001	**257.304**	0.006	-0.002	**392.196**	0.004	-0.094
**186.185**	-0.007	-0.059	**257.35**	0.010	0.014	**392.201**	0.040	0.011
**186.192**	0.008	-0.014	**257.36**	0.007	0.010	**392.21**	0.021	0.050
**186.217**	0.006	-0.011	**258.052**	0.017	0.028	**393.182**	0.002	-0.030
**186.454**	-0.051	0.010	**258.059**	0.073	0.096	**393.2**	0.012	0.017
**186.465**	-0.027	0.000	**258.068**	0.016	0.024	**396.171**	0.011	-0.012

**186.48**	-0.013	0.001	**258.074**	0.015	0.012	**396.183**	0.055	-0.097
**186.497**	-0.027	0.005	**258.081**	-0.029	0.002	**396.188**	0.007	-0.009
**186.512**	-0.141	0.021	**258.088**	-0.075	0.013	**396.26**	0.003	-0.008
**186.549**	-0.030	0.006	**258.099**	-0.007	0.032	**408.192**	0.009	0.011
**187**	-0.015	0.002	**258.11**	-0.014	-0.017	**408.211**	0.007	-0.008
**187.053**	0.007	-0.025	**258.115**	0.022	0.024	**408.219**	0.022	-0.033
**187.064**	-0.246	0.035	**258.128**	0.042	-0.083	**422.169**	0.012	0.017
**187.08**	0.052	0.039	**258.137**	0.049	0.080	**422.177**	0.010	0.014
**187.088**	0.114	0.037	**258.144**	-0.005	-0.038	**423.175**	0.007	0.005
**187.095**	0.046	-0.001	**258.149**	0.012	0.017	**433.27**	0.007	-0.009
**187.101**	-0.003	-0.042	**258.157**	0.027	0.021	**445.007**	0.010	0.012
**187.11**	0.015	0.003	**258.162**	-0.016	-0.005	**445.013**	0.028	0.010
**187.125**	0.012	0.011	**258.17**	0.005	-0.050	**445.019**	0.062	-0.060
**187.169**	-0.043	0.005	**258.178**	0.063	0.037	**445.024**	0.058	-0.031
**187.177**	-0.024	0.001	**258.187**	0.013	0.026	**445.03**	0.030	0.061
**187.185**	0.004	0.037	**258.215**	0.019	0.004	**445.036**	0.010	0.010
**187.201**	0.005	-0.007	**258.221**	0.011	0.016	**462.035**	0.005	-0.005
**187.209**	-0.155	0.016	**258.253**	0.033	0.034	**462.041**	0.010	-0.015
**187.217**	0.017	0.030	**258.262**	0.152	-0.024	**462.047**	0.009	-0.016
**187.225**	0.020	0.009	**258.287**	0.014	0.028	**462.053**	0.004	-0.008
**187.241**	0.007	-0.005	**258.295**	0.042	0.029	**470.094**	0.011	-0.012
**187.248**	0.017	0.034	**258.3**	0.078	-0.048	**470.102**	0.028	-0.035
**187.257**	-0.001	0.013	**258.325**	0.012	0.035	**470.111**	0.014	-0.012
**187.297**	-0.016	0.001	**258.334**	0.026	0.010	**508.269**	0.012	-0.006
**188.055**	0.022	0.018	**259.026**	0.007	0.005	**508.281**	0.012	-0.022
**188.061**	-0.041	0.062	**259.035**	-0.005	-0.038	**508.29**	0.041	-0.024
**188.07**	-0.118	0.018	**259.041**	0.010	0.006	**508.296**	0.005	-0.018
